# The potential use of mitochondrial ribosomal genes (12S and 16S) in DNA barcoding and phylogenetic analysis of trematodes

**DOI:** 10.1186/s12864-022-08302-4

**Published:** 2022-02-07

**Authors:** Abigail Hui En Chan, Naowarat Saralamba, Sompob Saralamba, Jiraporn Ruangsittichai, Urusa Thaenkham

**Affiliations:** 1 Department of Helminthology, Faculty of Tropical Medicine, Mahidol University, Bangkok, Thailand; 2 Department of Molecular Tropical Medicine and Genetics, Mahidol University, Bangkok, Thailand; 3 Mathematical and Economic Modelling (MAEMOD), Mahidol Oxford Tropical Medicine Research Unit, Faculty of Tropical Medicine, Mahidol University, Bangkok, Thailand; 4 Department of Medical Entomology, Faculty of Tropical Medicine, Mahidol University, Bangkok, Thailand

**Keywords:** Trematodes, Molecular identification, Molecular systematics, Mitochondrial ribosomal genes, Genetic marker, DNA barcoding

## Abstract

**Background:**

Genetic markers like the nuclear ribosomal RNA (rRNA) genes, internal transcribed spacer regions, mitochondrial protein-coding genes, and genomes have been utilized for molecular identification of parasitic trematodes. However, challenges such as the design of broadly applicable primers for the vast number of species within Digenea and the genetic markers’ ability to provide sufficient species-level resolution limited their utility. This study presented novel and broadly applicable primers using the mitochondrial 12S and 16S rRNA genes for Digenea and aimed to show their suitability as alternative genetic markers for molecular identification of orders Plagiorchiida, Echinostomida, and Strigeida.

**Results:**

Our results revealed that the mitochondrial 12S and 16S rRNA genes are suitable for trematode molecular identification, with sufficient resolution to discriminate closely related species and achieve accurate species identification through phylogenetic placements. Moreover, the robustness of our newly designed primers to amplify medically important parasitic trematodes encompassing three orders was demonstrated through successful amplification. The convenience and applicability of the newly designed primers and adequate genetic variation of the mitochondrial rRNA genes can be useful as complementary markers for trematode molecular-based studies.

**Conclusions:**

We demonstrated that the mitochondrial rRNA genes could be alternative genetic markers robust for trematode molecular identification and potentially helpful for DNA barcoding where our primers can be widely applied across the major Digenea orders. Furthermore, the potential of the mitochondrial rRNA genes for molecular systematics can be explored, enhancing their appeal for trematode molecular-based studies. The novelty of utilizing the mitochondrial rRNA genes and the designed primers in this study can potentially open avenues for species identification, discovery, and systematics in the future.

**Supplementary Information:**

The online version contains supplementary material available at 10.1186/s12864-022-08302-4.

## Background

Digenea Carus, 1863 is a subclass within Trematoda, and they represent one of the three major groups of helminths that parasitize humans and animals [1–5]. With more than 18,000 nominal species in over 2500 genera in Digenea, they are the most speciose group among the phylum Platyhelminthes [2]. The complex life-cycle of Digenea– usually requiring two intermediate hosts and definitive hosts, in addition to their highly ubiquitous nature contributes to their vast species diversity. Of these, a significant proportion is medically important to humans and animals, resulting in diseases that lead to mortality or morbidity. Schistosomiasis, Opisthorchiasis, and Fascioliasis are examples of notable diseases caused by parasitic trematodes that have plagued both humans and animals for many decades [5, 6].

The mainstay to disease diagnosis is the accuracy of species identification. Morphological-based methods using key diagnostic characters are the gold standard for trematode species identification [7]. However, the challenges with morphological-based methods are no stranger to taxonomists. The small size and soft bodies of adult trematodes, coupled with variations in characters due to morphological plasticity, hinder species identification accuracy [8, 9]. Different developmental stages of the trematode can also be found in various hosts, requiring the need to experimentally complete the trematode life-cycle to identify adults [10]. Moreover, parasite adaptation to different hosts from various geographical localities and climate changes can contribute greatly to genetic variation and speciation [11]. Higher numbers of cryptic species have been reported in trematodes than other groups of helminths, contributing to the complexity involved with morphological-based identification [12].

Molecular-based identification using molecular genetic markers has been widely applied to aid species identification of parasitic trematodes [8, 13, 14]. Guidelines and approaches for trematode molecular-based studies has been provided by Blasco-Costa et al., advocating the generation of more sequences for trematodes through genetic markers [15]. Genetic markers, either from the nuclear or mitochondrial DNA, provides an alternative source of characters for trematode species identification and phylogenetic studies. Many cryptic species have been discovered using genetic markers, unveiling greater diversity than morphological characters [8]. In addition, molecular-based identification provides a phylogenetic resolution to distinguish between morphologically similar species. The morphologically similar eggs belonging to *Opisthorchis* and Heterophyidae, along with the metacercariae and adults of *Opisthorchis viverrini* and *Opisthorchis lobatus* were successfully discriminated through molecular-based identification [16–18]. Thus, in the recent decade, the usefulness of molecular-based identification has appreciably accelerated the popularity of using genetic markers to aid in accurate trematode species identification.

For a genetic marker to be useful for species identification, it must possess sufficient sequence variation to discriminate between closely related species. In addition, the availability of primers that can be used to amplify many species within the target group of interest is essential, especially for the application of DNA barcoding [19, 20]. Genetic markers commonly used for trematode species identification and phylogenetic studies include the nuclear ribosomal RNA (rRNA) transcription unit (consisting of the rRNA genes and the internal transcribed spacers), the mitochondrial cytochrome *c* oxidase subunit 1 (*COI*) gene, and complete mitochondrial genomes [17, 21–26]. The nuclear 18S rRNA gene phylogeny provided by Olson et al. has served as the molecular bedrock for trematode molecular systematics and the primers are able to amplify many trematode species [24]. More recently, Pérez-Ponce de León and Hernández-Mena presented an updated trematode phylogeny using the nuclear rRNA genes [25]. However, it is widely known that the 18S rRNA gene possesses low sequence variation, especially among closely related species [21]. The lack of species-level resolution can limit their suitability for accurate species identification. Contrarily, the higher sequence variability of the mitochondrial *COI* gene and the internal transcribed spacer 2 (ITS2) region renders them useful for trematode species identification [17, 21, 22]. However, Moszczynska et al. showed that the high sequence variability of the platyhelminths *COI* sequence hinders successful amplification and limits the design of universal primers [10]. Consequently, primers for *COI* and ITS2 are usually designed to be genus or species-specific, targeting only a small number of species specific to the respective study [16, 22]. The limitations of the current genetic markers may restrict their utility to be widely applied for trematode species identification.

The mitochondrial 12S and 16S rRNA genes have been utilized for molecular studies of helminths, albeit not widely. Chan et al. revealed the potential of the mitochondrial ribosomal genes, where not only were they useful for nematode molecular systematics, but they also possess sufficient sequence variation to discriminate between closely related nematode species [27, 28]. The effectiveness of the 12S and 16S rRNA genes is further evidenced by applying the designed primers to amplify species across the four clades of parasitic nematodes. Among cestodes, species discrimination of *Mesocestoides* was achieved using the 12S rRNA gene [29]. In addition, Li et al. showed the effectiveness of the mitochondrial rRNA genes for inter-species discrimination among *Schistosoma*, proving their robustness for trematode species identification [30]. The robustness of the mitochondrial rRNA genes and their suitability for molecular studies of parasitic helminths cannot be disregarded; thus, the mitochondrial rRNA genes could also be promising for trematodes of medical importance.

To, therefore, show the suitability of the mitochondrial rRNA genes as alternative genetic markers for parasitic trematodes, we provide evidence to show the genetic markers’ robustness for species identification. We demonstrate that the mitochondrial rRNA genes contain sufficient sequence variation and designed primers encompassing the three orders (Plagiorchiida, Echinostomida, and Strigeida) of parasitic trematodes within Digenea for species discrimination. Using the mitochondrial rRNA genes, we aimed to provide alternate genetic markers and primers conveniently applied for trematode molecular identification of the medically important groups.

## Results

### Molecular phylogenetics of representative trematodes using 12S and 16S rRNA genes

Of the 26 representative trematode species belonging to the three orders sampled in this study, they were accurately identified using the mitochondrial 12S and 16S rRNA genes and appropriately placed on the phylogenetic trees. All the representative trematode species were successfully amplified, including cercaria, metacercaria, and adult stages.

#### Plagiorchiida

**Fig. 1:**
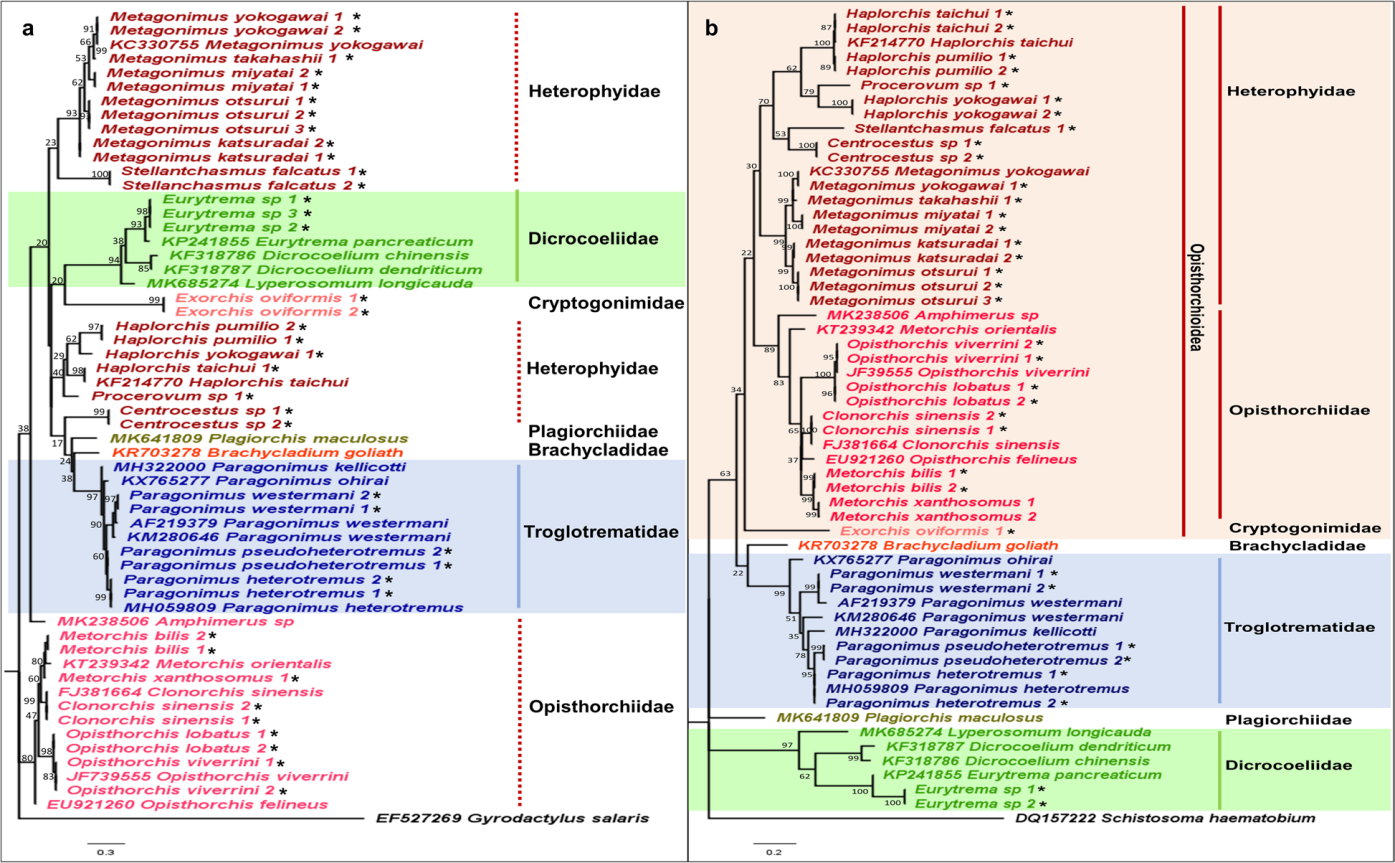
Maximum likelihood phylogenetic trees of (**a**) 12S rRNA gene (GTR + G) and (**b**) 16S rRNA gene (HKY + G) for order Plagiorchiida. Numbers at nodes indicate bootstrap values. Representative sequences generated from this study are indicated with an ‘*’. The superfamilies/families that were recovered as monophyletic are highlighted

Twenty-one representative species belonging to families Opisthorchiidae, Heterophyidae, Cryptogonimidae, and Troglotrematidae were included for analysis in Plagiorchiida. As presented in Fig. 1a and b, the phylogenetic trees inferred using the mitochondrial 12S and 16S rRNA genes showed that the species were accurately identified based on the clustering with the appropriate reference sequences. The 12S and 16S rRNA gene phylogenies showed a clear distinction between trematode species known to be closely related. For example, *Paragonimus heterotremus* and *Paragonimus pseudoheterotremus* were differentiated based on both the 12S and 16S rRNA phylogenetic trees. As shown in Table 1, genetic distances revealed that the difference between the two species of *Paragonimus* was 2.9 and 3.9% using the 12S and 16S rRNA genes, respectively. Contrarily, the two species were not able to be differentiated using the nuclear 18S rRNA gene, with no difference in the sequences, as indicated in Table 1 and Additional file 1: Fig. S1. Genetic distances between *P. heterotremus* and *P. pseudoheterotremus* were 0.5 and 1.9% for the nuclear 28S rRNA gene and the ITS2 region, respectively, and the values are comparatively lower than the genetic distances obtained using the mitochondrial rRNA genes.

**Table 1:** Percentage of nucleotide difference between species (inter-species genetic distance) based on each genetic marker, expressed in percentage

		12S	16S	***COI***	18S	28S	ITS2
**Plagiorchiida**	Inter-species (Opisthorchiidae)	9.0%	10.0%	17.0%	0%	3.0%	5.0%
	*O. viverrini*/*O. lobatus*	1.2%	1.2%	NA	NA	NA	0%
	Inter-species (Troglotrematidae)	6.0%	9.0%	16.0%	1.0%	3.0%	7.0%
	*P. pseudoheterotremus*/*P. heterotremus*	2.9%	3.9%	NA	0%	0.5%	1.9%
**Echinostomida**	Inter-species (Fasciolidae)	13.0%	9.0%	14.0%	1.0%	4.0%	13.0%
	*F. gigantica*/*F. hepatica*	11.5%	5.9%	9.2%	0.3%	0.5%	0.4%
**Strigeida**	Inter-species (Schistosomatidae)	15.0%	17.0%	22.0%	2.0%	7.0%	15.0%
	*S. japonicum*/*S. mekongi*	5.2%	8.1%	15.3%	0.4%	2.7%	5.1%

NA indicates not applicable due to no sequence available

Likewise, within the family Opisthorchiidae, the 12S and 16S rRNA genes showed sufficient variation to differentiate between *Opisthorchis* spp., *Clonorchis sinensis*, and *Metorchis* spp. The result contrasts with the nuclear 18S rRNA gene phylogeny, where species within Opisthorchiidae (except *Amphimerus ovalis*) were not successfully differentiated. Genetic distances within the family Opisthorchiidae further support this finding, and the results revealed no sequence variation using the 18S rRNA gene within Opisthorchiidae. On the other hand, genetic distances of 9.0 and 10.0% were observed using the 12S and 16S rRNA genes within Opisthorchiidae, proving that the mitochondrial rRNA genetic markers provide sufficient sequence variation to differentiate species within the family Opisthorchiidae. Additionally, genetic distances between the closely related *O. viverrini* and *O. lobatus* using the 12S and 16S rRNA genes were 1.2%, whereas the ITS2 region could not discriminate between the two closely related species.

Comparing the mitochondrial 12S and 16S rRNA gene phylogenies based on successfully recovered families as monophyletic, the 16S rRNA gene phylogeny provided better resolution than the 12S rRNA gene phylogeny. All representative families that contained more than one species were recovered as monophyletic in the 16S rRNA gene phylogeny, whereas only two families– Troglotrematidae and Dicrocoeliidae, were recovered as monophyletic in the 12S rRNA gene phylogeny. Additionally, superfamily Opisthorchioidea, which comprises families Opisthorchiidae, Heterophyidae, and Cryptogonimidae, was recovered as monophyletic, albeit the low bootstrap support.

#### Echinostomida

**Fig. 2:**
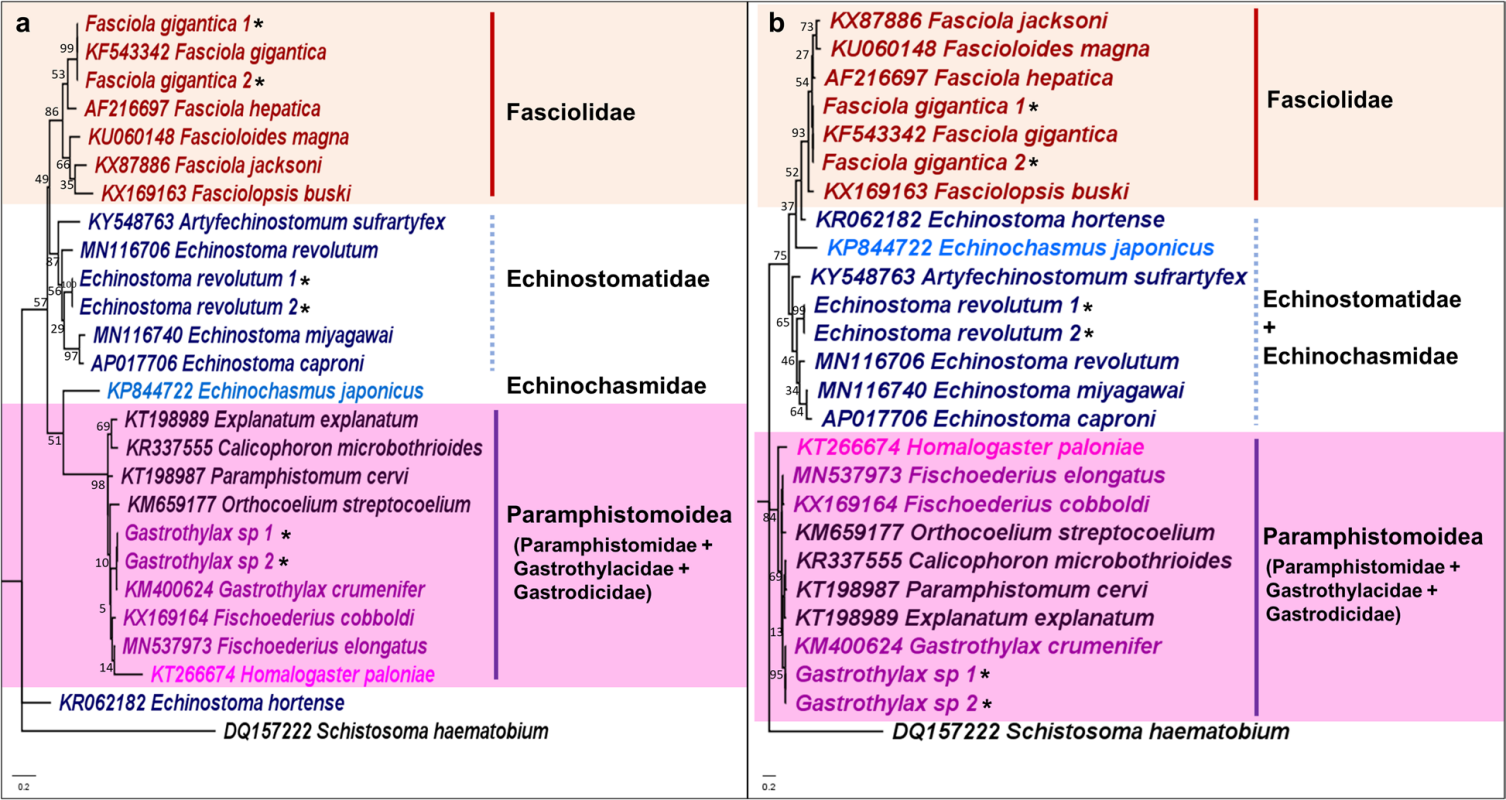
Maximum likelihood phylogenetic trees of (**a**) 12S rRNA gene (GTR + G) and (**b**) 16S rRNA gene (GTR + G) for order Echinostomida. Numbers at nodes indicate bootstrap values. Representative sequences generated from this study are indicated with an ‘*’. The families/superfamilies that were recovered as monophyletic are highlighted

The three representative species (*F. gigantica*, *Echinostoma revolutum*, and *Gastrothylax* sp.) belong to Fasciolidae, Echinostomatidae and Gastrothylacidae were used for analysis. As shown in Fig. 2a and b, the representative species were accurately identified and clustered together with their respective reference sequences in both the 12S and 16S rRNA phylogenetic trees. The closely related *F. hepatica* and *F. gigantica* were also clearly distinguished using the 12S and 16S rRNA genes. Genetic distances between the two species using the 12S and 16S rRNA genes were 11.5 and 5.9%, respectively, and the values were comparatively higher than the three nuclear DNA genetic markers (Table 1). Similarly, inter-species genetic distances within the family Fasciolidae using the mitochondrial rRNA genes were also higher when compared with the nuclear rRNA genes.

Between the phylogenetic trees of the mitochondrial 12S and 16S rRNA genes, the tree topologies were similar. Only Fasciolidae was recovered as monophyletic with strong BS support, whereas Echinostomatidae, Paramphistomidae, and Gastrothylacidae were not monophyletic. The close relationship between families Fasciolidae and Echinstomatidae was supported in both phylogenetic trees. Also, the monophyly of superfamily Paramphistomoidea, comprising Gastrothylacidae, Paramphistomidae, and Gastrodicidae, was supported by the 12S and 16S phylogenetic trees. The nuclear 28S rRNA gene phylogeny, presented in Additional file 1: Fig. S2, recovered the four families as monophyletic, showing better resolution at higher taxonomic levels than the 12S and 16S rRNA genes.

#### Strigeida

**Fig. 3:**
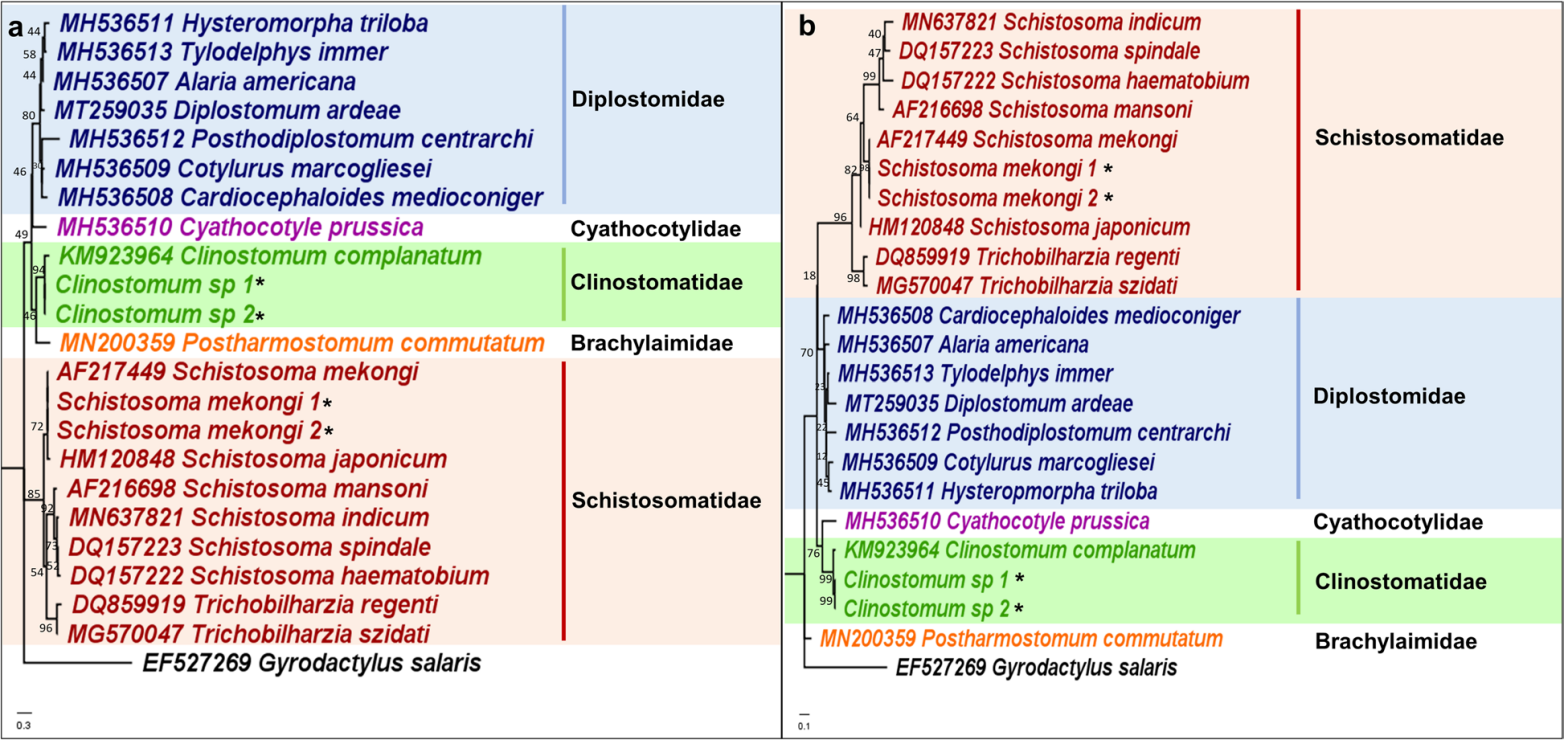
Maximum likelihood phylogenetic trees of (**a**) 12S rRNA gene (HKY + G) and (**b**) 16S rRNA gene (GTR + G) for order Strigeida. Numbers at nodes indicate bootstrap values. Representative sequences generated from this study are indicated with an ‘*’. The families that were recovered as monophyletic are highlighted

The two representative species of Schistosomatidae and Clinostomatidae were accurately placed on the 12S and 16S rRNA gene phylogenetic trees, as shown in Fig. 3a and b. The two closely related species, *Schistosoma mekongi,* and *Schistosoma japonicum,* were also distinguished based on the phylogenies. Supported by genetic distance values, genetic distances between *S. mekongi* and *S. japonicum* using the 12S and 16S rRNA genes were 5.2 and 8.1%, respectively (Table 1). Comparing with the genetic distances of the nuclear DNA genetic markers between *S. mekongi* and *S. japonicum*, distances for the nuclear 18S rRNA gene were 0.4 and 2.7% for the nuclear 18S and 28S rRNA genes, and 5.1% for the ITS2 region. Like the results from the other closely related species mentioned earlier, higher levels of genetic variation can be observed using the mitochondrial rRNA genetic markers compared to the nuclear DNA genetic markers. Additionally, both 12S and 16S rRNA gene phylogenies were able to recover families Schistosomatidae, Clinostomatidae, and Diplostomidae as monophyletic with strong BS support, similar to the nuclear rRNA genes (Additional file 1: Fig. S3).

## Discussion

Our findings demonstrated the robustness of the mitochondrial 12S and 16S rRNA genes for trematode species identification using the representative species belonging to the three trematode orders. Firstly, the representative species were appropriately placed on the phylogenetic trees, and genetic distances were sufficient even to discriminate between closely related species. Secondly, the suitability of the mitochondrial 12S and 16S genes is further substantiated through the ability of the designed primers to amplify parasitic trematode species of medical importance encompassing the three orders.

### Accurate taxonomic assignments and species discrimination using the mitochondrial rRNA genes

Representative trematodes were used to determine the suitability of the mitochondrial rRNA genes for the molecular identification of trematodes. Only a few studies have utilized the mitochondrial rRNA genes for species identification of helminths to date. Our results indicate that both the 12S and 16S rRNA genes are suitable for trematode molecular identification, as revealed through accurate taxonomic assignments through phylogenetic placements, genetic distances between closely related species higher than the nuclear DNA genetic markers, and comparable inter-species genetic distances to the mitochondrial *COI* gene.

Of the 26 representative species of trematodes encompassing 10 families within three orders sampled in this study, all were accurately placed on the phylogenetic trees with the corresponding reference sequences. In instances where no reference sequence was available for the specific representative species, they were still correctly assigned to either the genus or family level. Clear clade distinctions in the phylogenetic trees were also observed between closely related species. Additionally, an adult specimen that was initially morphologically identified as *Eurytrema* sp. was grouped with *Gastrothylax* in the phylogenetic trees. Morphological re-examination allowed us to correctly re-identify the specimen as *Gastrothylax*, with the presence of a prominent ventral pouch extending posteriorly to the level of testes [5]. As both *Eurytrema* and *Gastrothylax* utilize cattle as definitive hosts, misidentification due to similar hosts can occur. The accuracy of taxonomic assignments, coupled with the potential to identify unknowns allowing for the confirmation of species identification, show the immense potential of the mitochondrial rRNA genes as alternative genetic markers for trematodes.

Among the various genetic markers, the nuclear ITS2 region is a genetic marker commonly used for the molecular identification of trematodes due to its ability for species discrimination. Successful species discrimination using ITS2 was achieved between the closely related *Dicrocoelium chinensis* and *Dicrocoelium dentriticum*, and among closely related species in families Schistosomatidae and Diplostomatidae [31, 32]. Our findings corroborate previous studies, showing that the ITS2 region possesses sufficient genetic variation to discriminate between species. However, we observed that genetic variations between closely related species were lower than the mitochondrial DNA genetic markers. Previous studies have also revealed lower levels of sequence variation among closely related trematode species using the ITS2 region than the mitochondrial genes [33, 34]. Despite the comparable inter-species genetic distances of the ITS2 region to the mitochondrial DNA genetic markers, the comparatively lower sequence variation between closely related trematodes could limit the robustness of the ITS2 region for molecular identification among cryptic lineages, conferring an advantage to the mitochondrial rRNA genes. Aside from the ITS2 region, the nuclear rRNA genes possess smaller inter-species genetic distances when compared to the mitochondrial rRNA genes.

Although the nuclear rRNA genes are established genetic markers for molecular-based studies at a higher taxonomic level, the low genetic variation could be problematic for species identification, especially among cryptic lineages. The low levels of genetic variation observed for the nuclear rRNA genes are no surprise, as various studies have also indicated the limited usability of the nuclear rRNA genes for species identification [13, 21]. In particular, Kang et al. found no difference in the partial 18S rRNA gene sequences between *Opisthorchis viverrini*, *Opisthorchis felineus,* and *Clonorchis sinensis* [21]. Clearly, as compared to the nuclear DNA genetic markers, the mitochondrial rRNA genes of 12S and 16S can be alternative genetic markers to the nuclear rRNA genes and ITS2 region that is robust for trematode molecular identification.

The mitochondrial *COI* gene, also known as the ‘DNA barcode’ of choice among other organisms, is highly popular for trematode molecular identification [19]. The *COI* gene possesses sufficient genetic variation to discriminate between species, including cryptic lineages. Among others, examples include molecular identification of species within the family Heterophyidae, Opisthorchiidae, and the *Paragonimus heterotremus* complex [17, 22, 33, 35]. In our study, inter-species genetic distances among the three mitochondrial DNA genetic markers are comparable. Having comparable genetic distances and discriminating closely related species demonstrates that the mitochondrial rRNA genes can hold a candle to the famous *COI* gene, indicating their suitability for trematode molecular identification. Our findings corroborate previous studies, as successful discrimination of nematode species within the *Angiostrongylus cantonensis* lineage was also attained using these two genetic markers [28]. More recently, the suitability of the mitochondrial 12S and 16S rRNA genes for molecular identification of parasitic trematodes, cestodes, and nematodes were ascertained through comparisons with other genetic markers [36]. Although the mitochondrial rRNA genes are generally the most conserved of the genes in the mitochondrial genome, here we provide evidence of the ability of the mitochondrial genes as alternative genetic markers for molecular identification of trematodes, instead of the commonly used mitochondrial protein-coding genes. The robustness of the mitochondrial 12S and 16S rRNA genes for molecular identification of trematodes is indicative of their suitability as alternative genetic markers, which can be valuable for species identification of trematodes.

### Utility of developed 12S and 16S primers to cover a wide range of species in Digenea

For molecular identification, besides the ability of the genetic marker possessing sequence variation to discriminate between species, another key aspect lies in the success of the developed primer to amplify many species within the target group of organisms [37]. Having a primer that can target many species within Digenea is important, facilitating the ease of DNA barcoding studies where a ‘universal’ primer and genetic marker are used [19, 20]. Our mitochondrial 12S and 16S primers developed in this study can cover the three orders within Digenea that contain trematodes of medical importance to humans and animals, being able to amplify 26 species from actual specimens. Furthermore, all the representative specimens consisting of various life-cycle stages were successfully amplified from both primer sets, showing high sensitivity by amplifying minute gDNA amounts.

The current ‘universal’ primers that have been utilized for molecular studies for trematodes include primers for the mitochondrial *COI* and *NAD1* gene, the ITS2 region, and the nuclear 18S rRNA gene [38–43]. However, limitations restrict their robustness for trematode molecular identification. The species-level resolution of the 18S rRNA gene is problematic for molecular identification due to low levels of genetic variation between some species. Moreover, a longer amplicon is usually required for the 18S rRNA gene due to lesser phylogenetically informative sites than other genetic markers with higher levels of genetic variation. Thus, the 18S primers, despite the ability to amplify a wide variety of species for trematodes, are limited in their use for species identification due to low levels of genetic variation.

Conversely, the mitochondrial *COI* gene, *NAD1* gene, and the ITS2 region, with their higher levels of genetic variation, are helpful for molecular identification [17, 22, 31, 32]. The primers for the ITS2 region usually target the conserved flanking sequences of the 5.8S and 28S rRNA gene, allowing the amplification of the entire ITS2 region [13]. However, repeat sequences and high sequence variability in the ITS2 region complicate the downstream processes of sequence and phylogenetic analysis. Consequently, genus- or species-specific primers are designed instead to target the specific group of trematodes studied.

Degenerate *COI* primers targeting the DNA barcoding region were developed by Van Steenkiste et al. [42]. Also, the *COI* JB3/JB4.5 primers developed by Bowles et al. were successfully utilized for molecular identification of various groups of trematodes [22, 38, 43]. However, the high sequence variation hinders the successful amplification of trematodes using the available *COI* primers [10, 43]. Although Van Steenkiste et al. showed 100% PCR amplification success using the degenerate *COI* primers, troubleshooting and the use of three set of primers was required to obtain 100% success [42]. The *NAD1* gene is popular among family Echinostomatidae, and successful molecular discrimination among the morphologically similar *Echinostoma revolutum* species complex have been achieved [43–47]. Additionally, the *NAD1* gene showed higher levels of inter species variation compared to the *COI* gene within *Echinostoma* and among trematodes [36, 43]. Similar to the *COI* gene, although they are useful for species discrimination, the high sequence variatblity can be a limiting factor hindering amplification of broad species range. Thus, despite these three genetic markers possessing sufficient genetic variation helpful for species identification, their use is limited due to high sequence variability.

Compared to the *COI* and *NAD1* genes, the slightly more conserved sequences of the mitochondrial 12S and 16S rRNA genes allowed us to design ‘universal’ primers for a wide range of trematode species. This property was evidenced through the successful amplification of trematode species in this study. Moreover, aside from trematodes, the primers designed in this study can amplify medically important cestode species (results not shown). Additionally, the mitochondrial rRNA genes have higher levels of sequence variation than the 18S rRNA gene, allowing successful discrimination of closely related species. Despite being short in length, genetic variation was present for the partial amplicons of the mitochondrial rRNA genes with more phylogenetically informative sites, allowing for species discrimination.

Moreover, the short amplicon length increases the chances of successful PCR amplification, rendering them useful to amplify archived specimens, especially for DNA barcoding studies. Similarly, primers targeting parasitic nematode species of medical importance were designed and evaluated for molecular systematics and identification, showing the possibility of utilizing the mitochondrial rRNA genes for DNA barcoding of nematodes [27]. Although the current DNA barcode of choice is the mitochondrial *COI* gene, the potential of the mitochondrial rRNA genes for DNA barcoding of trematodes cannot be undermined. Trematode DNA barcoding can be possible with the developed primers in this study that targets a broad range of species within Digenea and having an adequate taxonomic resolution to the species level.

### Additional advantages of the mitochondrial rRNA genes as genetic markers for trematodes

The value of the mitochondrial rRNA genes as alternative genetic markers for molecular studies of trematodes is further enhanced by being a proxy for complete mitochondrial genomes and their potential for molecular systematics. Phylogenetic studies utilizing the complete mitochondrial genomes have gained popularity over the years, with the advancements in sequencing techniques, and have since been a valuable source of information for phylogenetic studies [23, 48–51]. However, resources for sequencing complete mitochondrial genomes might not be readily available, and laboratory capabilities are usually budget-dependent. Here, our results using the partial 12S and 16S rRNA gene sequences revealed a similar clade arrangement to mitochondrial genome phylogenies despite being shorter in sequence length. The good resolution for trematode phylogenetic studies provided by the partial 12S and 16S rRNA genes can serve as a proxy for mitochondrial genomes. Within order Strigeida, previous studies using mitochondrial genomes support a sister relationship between Clinostomidae and Diplostomidae, with Schistosomatidae then sister to both [52–54]. Our 12S rRNA gene phylogeny revealed a similar phylogeny, where Clinostomatidae shows a closer relationship to Diplostomidae than Schistomsomatidae. Likewise, relationships within order Plagiorchiida showed a similar phylogeny to mitochondrial genomes [55, 56].

In our 16S rRNA gene phylogeny, superfamily Opisthorchioidea (with families Opisthorchiidae, Heterophyidae, and Cryptogonimidae as representatives) showed a closer relationship to Troglotrematidae as compared to Dicrocoeliidae. Using complete mitochondrial genomes, the close relationship of Opisthorchioidea and Troglotrematidae was also supported, with Dicrocoeliidae as a sister group. The robustness of the partial mitochondrial rRNA genes for phylogenetic studies is evidenced through the resolution of relationships obtained compared with mitochondrial genome phylogenies. Although the partial 12S and 16S rRNA gene sequences are approximately 370 bp in length, having a short length with sufficient phylogenetic resolution increases the genetic markers’ practicality and convenience for molecular-based studies, more advantageous than sequencing complete mitochondrial genomes, especially when resources are limited.

In addition to the suitability of the mitochondrial 12S and 16S rRNA genes for trematode molecular identification, these two genetic markers also carry the potential for molecular systematics studies of trematodes. First, the phylogenies obtained from the partial 12S and 16S rRNA gene sequences showed a similar clade arrangement to mitochondrial genome phylogenies. Second, the primers developed in this study can amplify a broad taxonomic range of trematodes. Third, the genetic markers are not saturated for nucleotide substitutions, rendering them useful for molecular systematics. Fourth, the availability of full-length 12S and 16S rRNA gene sequences in reference databases due to many complete mitochondrial genomes being sequenced allows for many taxa comparisons. Last, although the phylogenetic relationships obtained with the 12S and 16S rRNA genes were not congruent with nuclear phylogenies, the phylogenies obtained using the concatenated sequences of the mitochondrial rRNA genes with nuclear rRNA genes were similar with nuclear phylogenies. The concatenated gene phylogenies are presented in Additional file 2: Figs. S1 to S3.

For example, within order Strigeida, the relationship between families Clinostomidae, Diplostomidae, and Schistosomatidae have been debatable due to differences in phylogenies between mitochondrial genomes and nuclear DNA genetic markers. Nuclear phylogenies support a sister relationship between Clinostomidae and Schistosomatidae, with Diplostomidae then placed sister to the two, whereas mitochondrial phylogenies support a closer relationship between Clinostomidae and Diplostomidae [52–54, 57]. By concatenating the nuclear rRNA genes with either the mitochondrial 12S and 16S rRNA genes, a sister relationship resulted between Clinostomatidae and Schistosomatidae, supporting nuclear phylogenies. An increase in the number of families recovered as monophyletic also resulted with orders Echinostomida and Plagiorchiida through the concatenated mitochondrial and nuclear rRNA gene phylogenies, as compared to the mitochondrial rRNA gene phylogenies. The nuclear rRNA genes are indisputably robust genetic markers for trematode molecular systematics but might not contain adequate resolution at the species level [24]. The value of concatenating the nuclear rRNA genes with the mitochondrial rRNA genes can potentially increase species-level taxonomic resolution. The use of genetic markers from two different loci has been suggested and utilized by various studies, but they have rarely been combined to improve phylogenetic resolution for molecular systematics [15, 31, 52, 57–59]. Our findings, therefore, demonstrate the potential of combining both the nuclear and mitochondrial DNA as an alternative genetic marker for trematode molecular systematics. However, as we obtained the sequences for the nuclear rRNA genes from the NCBI database, investigations on the full potential of using concatenated markers for trematode molecular systematics will have to be performed.

### Limitations

Analysis for this study was subjected to the availability of complete mitochondrial genomes present in reference databases. Additionally, the accuracy of molecular identification is subjected to the accuracy of the reference sequences in the NCBI database, along with morphological identification. Low nodal support was observed for some families (e.g. Heterophyidae in the 16S rRNA gene phylogeny), possibility due to the short amplicon length of the sequence generated. As the mitochondrial 12S and 16S genes are not widely utilized for trematodes, the limited number of sequences present in reference databases limits the scale of comparision and primer design across the whole of Digenea. More data is also required in the future to determine the full utility of the 12S and 16S rRNA genes for DNA barcoding. However, the primers targeting the mitochondrial rRNA genes in this study can serve as a stepping stone for the generation of more sequences to determine intraspecific variation and the DNA barcoding gap.

## Conclusions

Molecular identification of trematodes using the mitochondrial 12S and 16S rRNA genes was achieved, including the successful discrimination of closely related species. The suitability of the mitochondrial rRNA genes for trematode molecular identification was demonstrated through the robustness of the genetic marker and the ability of the developed primers to amplify medically important parasitic trematodes encompassing three orders. We have also revealed the advantages of using the mitochondrial rRNA genes as genetic markers for DNA barcoding studies, with adequate genetic variation for species identification coupled with primers conveniently applied. The mitochondrial rRNA genes can be complementary to other genetic markers for trematode molecular identification, serving as useful alternatives. Additionally, the resolution power of these two genetic markers as a proxy for complete mitochondrial genomes and molecular systematics cannot be overlooked. Future research includes expanding the taxa studied using the mitochondrial rRNA genes, building up a comprehensive reference database enhancing the quality of molecular identification, and evaluating the possibility of utilizing these genetic markers for DNA barcoding.

## Materials and methods

### Taxon sampling

#### Representative trematodes for mitochondrial 12S and 16S rRNA gene sequences

Representative trematodes belonging to the three orders used for the study are part of the archived specimens in the Department of Helminthology, Faculty of Tropical Medicine, Mahidol University, Bangkok, Thailand. They were previously collected from intermediate and definitive hosts, preserved in 70% ethanol, and stored at − 20 °C. Individual trematodes were morphologically identified, primarily to species level, based on morphological characters with taxonomic keys.

Using the complete mitochondrial genomes of trematodes available on the NCBI database (http://www.ncbi.nlm.nih.gov), full-length mitochondrial 12S and 16S rRNA gene sequences were obtained for each trematode species. A total of 107 sequences encompassing 71 species across 16 families were used for analysis together with our representative sequences. All DNA sequences used are listed in Additional file 3: Table S1.

#### Representative trematode sequences of nuclear 18S and 28S rRNA genes, ITS2 regions, and mitochondrial COI gene

Full-length and partial sequences of the nuclear 18S and 28S rRNA genes, the ITS2 region, and the full-length mitochondrial *COI* gene sequences were mined from the NCBI database (Additional file 3: Table S1). The sequences of four other genetic markers were obtained to compare with the mitochondrial 12S and 16S rRNA genes to measure the genetic markers’ robustness for molecular identification. To the best extent possible, the same trematode species were selected for each genetic marker.

### Molecular analyzes

#### DNA extraction

Individual trematode samples were placed into 1.7 ml microcentrifuge tubes and washed thoroughly with sterile distilled water. For larger-sized trematodes, a small section of the sample was removed for DNA extraction, and the remainder of the sample was stored back in 70% ethanol for future analysis. In cases where the trematodes were too small, individuals were directly placed into the DNA lysis buffer. Total genomic DNA was isolated from each individual using the Geneaid genomic DNA mini kit (Geneaid Biotech Ltd., Taipei, Taiwan) following the manufacturer’s recommendations.

#### Primer design

Primers for trematode mitochondrial 12S and 16S rRNA genes were designed using the DNA sequences obtained from NCBI (Additional file 3: Table S1). DNA sequences were aligned using ClustalX 2.1 [60], and conserved regions for possible primer binding sites were manually checked with Bioedit 7.0 [61]. The primers for both genes were then designed at the conserved regions. The GC content, melting temperature, and hairpin formation were predicted and calculated by OligoCalc version 3.27 (http://biotools.nubic.northwestern.edu/OlicoCalc.html) [62]. In silico PCR was performed using FastPCR [63] to check the amplicon size and ensure that the newly designed 12S and 16S primers can successfully amplify the representative trematode species. A gradient PCR was performed to optimize PCR conditions and annealing temperature for each primer. The nucleotide sequences of the primers, together with the amplicon size and thermocycling conditions, are provided in Table 2.

**Table 2:** Designed primer sequences with the respective annealing temperature and amplicon size for gene amplification

Target gene	Primer name	Sequence (5′-3′)	Annealing temperature (°C)	Amplicon size (bp)
12S rRNA	Tre12S-F	GTGCCAGCADYYGCGGTTA	55	371
	Tre12S-R	AGCAGCAYATHGACCTG		
16S rRNA	CesTre16S-F	GTGYDAAGGTAGSATAAT	56	379
	CesTre16S-R	CCGGTYTYAACTCARCTCAT		

#### PCR and DNA sequencing

The partial mitochondrial 12S and 16S rRNA genes were amplified in a T100™ thermocycler (Bio-Rad, California, USA) with the primers in Table 2. Each 30 μl reaction contained 15 μl of 2X i-Taq™ master mix (iNtRON Biotechnology, Gyeonggi, South Korea), 0.1 μM of each primer, and 1 ng/μl of DNA. The thermocycling profiles were 94 °C for 2 min of initial denaturation; 35 cycles of 94 °C for 30s, 55 °C or 56 °C for 1 min, and 72 °C for 2 min; followed by a final extension step at 72 °C for 5 min. PCR amplicons were visualized on 1% agarose gel stained with GelRed® (Thomas Scientific, New Jersey, USA). Successful amplicons were purified with Geneaid PCR Purification Kit (Geneaid Biotech Ltd., Taipei, Taiwan) following the manufacturer’s recommendations. Purified DNA products were sequenced by a commercial company (Macrogen, Seoul, South Korea) on an automated Sanger sequencer using the primers for PCR amplification. The partial mitochondrial 12S and 16S rRNA gene sequences generated in this study were deposited in the NCBI database (12S rRNA gene sequences: MZ331635–MZ331683; 16S rRNA gene sequences: MZ331595–MZ331634, MZ345698–MZ345705).

### Phylogenetic analysis

#### Analysis of representative trematodes for mitochondrial 12S and 16S rRNA gene sequences

Electropherograms of the partial 12S and 16S rRNA gene sequences were manually checked using Bioedit 7.0 [61]. The sequences were then aligned with the reference sequences obtained from the NCBI database (Additional file 3: Table S1) using ClustalX 2.1 [60]. We performed sequence alignment and phylogenetic analysis for each of the three trematode orders (Plagiorchiida, Echinostomida, and Strigeida) separately. Taxonomic grouping to the three orders was based on the classification by the Keys to the Trematoda and Olson et al. [2–4, 24]. The aligned sequences were manually checked, and phylogenetic analysis using the maximum likelihood (ML) method was performed with MEGA X [64]. ML analysis was conducted using the best-fit nucleotide substitution model with 1000 bootstrap iterations (BS) for tree topology support. BS of > 70% was considered to be strong [65]. *Gyrodactylus salaris* and *Schistosoma haematobium* were used as outgroups to root the phylogenetic trees. The phylogenetic trees were visualized and labeled with FigTree 1.3.1 [66].

#### Comparison of inter-species genetic distances, phylogenetic analysis, and testing for nucleotide substitution saturation among genetic markers

To determine the robustness of the mitochondrial 12S and 16S rRNA genes for trematode molecular identification, we analyzed inter-species genetic distances and reconstructed phylogenetic trees to compare the six genetic markers. The mitochondrial 12S and 16S rRNA genes were compared with the nuclear 18S and 28S rRNA genes, ITS2 region, and the mitochondrial *COI* gene.

Pair-wise inter-species genetic distances using *P*-distance as the model were obtained for each genetic marker with MEGA X [64], where the calculated genetic distances were categorized to derive the average inter-species genetic distance. Genetic distances between selected closely related species were also obtained. We selected representative families and closely related species for each order for comparison between the genetic markers. The representative species for inter-species are: within family Opisthorchiidae and Troglotrematidae for Plagiorchiida, within Fasciolidae for Echinostomida, and within Schistosomatidae for Strigeida. The selected closely related species are: *O. lobatus* and *O. viverrini*, *Paragonimus heterotremus* and *Paragonimus pseudoheterotremus*, *Fasciola hepatica* and *Fasciola gigantica*, *Schistosoma japonicum* and *Schistosoma mekongi*.

ML phylogenetic trees were reconstructed for the nuclear 18S and 28S rRNA genes, ITS2 region, and the mitochondrial *COI* gene with the method described in the previous section. The genetic markers were tested for nucleotide substitution saturation using DAMBE 6 [67], where saturation was based on the values of *Iss* (simple index of substitution saturation) and *Iss.c* (critical *Iss* value), with *Iss* < *Iss.c* indicating that the genetic marker was not saturated and vice versa.

## Supplementary Information

**Additional file 1: Figure S1 to S3.** Maximum likelihood phylogenetic trees for the nuclear 18S rRNA gene, 28S rRNA gene, ITS2 region, and the mitochondrial *COI* gene.

**Additional file 2: Figure S1 to S3.** Maximum likelihood phylogenetic trees for the concatenated nuclear rRNA and mitochondrial rRNA genes.

**Additional file 3: Table S1.** NCBI sequences generated in this study and trematode species used for phylogenetic analysis.

## Data Availability

All data generated or analyzed during this study are included in the published article and its supplementary information files. The newly generated sequences were deposited in the GenBank database under the accession numbers MZ331635–MZ331683, MZ331595–MZ331634, and MZ345698–MZ345705.

## Abbreviations

PCR: Polymerase chain reaction
NCBI: National Center for Biotechnology Information
rRNA: ribosomal RNA
ITS2: internal transcribed spacer 2
*COI*: cytochrome *c* oxidase subunit 1
ML: Maximum likelihood
BS: Bootstrap
