# Supplementary material for: The potential use of mitochondrial ribosomal genes (12S and 16S) in DNA barcoding and phylogenetic analysis of trematodes
Source: BMC Genomics. 2022 Feb 7;23:104. doi: 10.1186/s12864-022-08302-4 (PMC8822746; doi:10.1186/s12864-022-08302-4)
Supplement: Supplementary file 1 — Additional file 1: Figure S1 to S3. Maximum likelihood phylogenetic trees for the nuclear 18S rRNA gene, 28S rRNA gene, ITS2 region, and the mitochondrial COI gene. [file 12864_2022_8302_MOESM1_ESM.docx]

**Additional file 1:** Maximum likelihood phylogenetic trees for the nuclear 18S rRNA gene, 28S rRNA gene, ITS2 region, and the mitochondrial *COI* gene

**
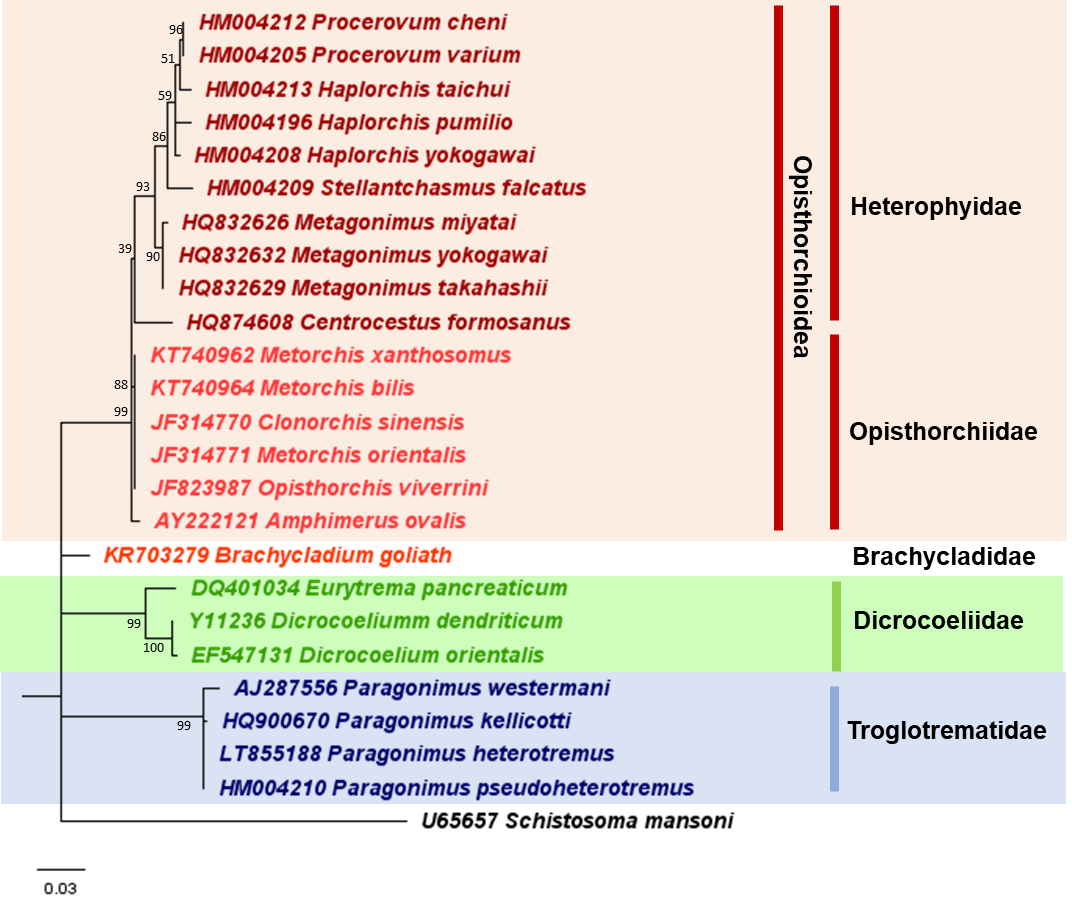
**

**Fig. S1a** Maximum likelihood phylogenetic tree (K2P+G) of the 18S rRNA gene for order Plagiorchiida

Numbers at nodes indicate bootstrap values. The superfamilies/families that were recovered as monophyletic are highlighted

**
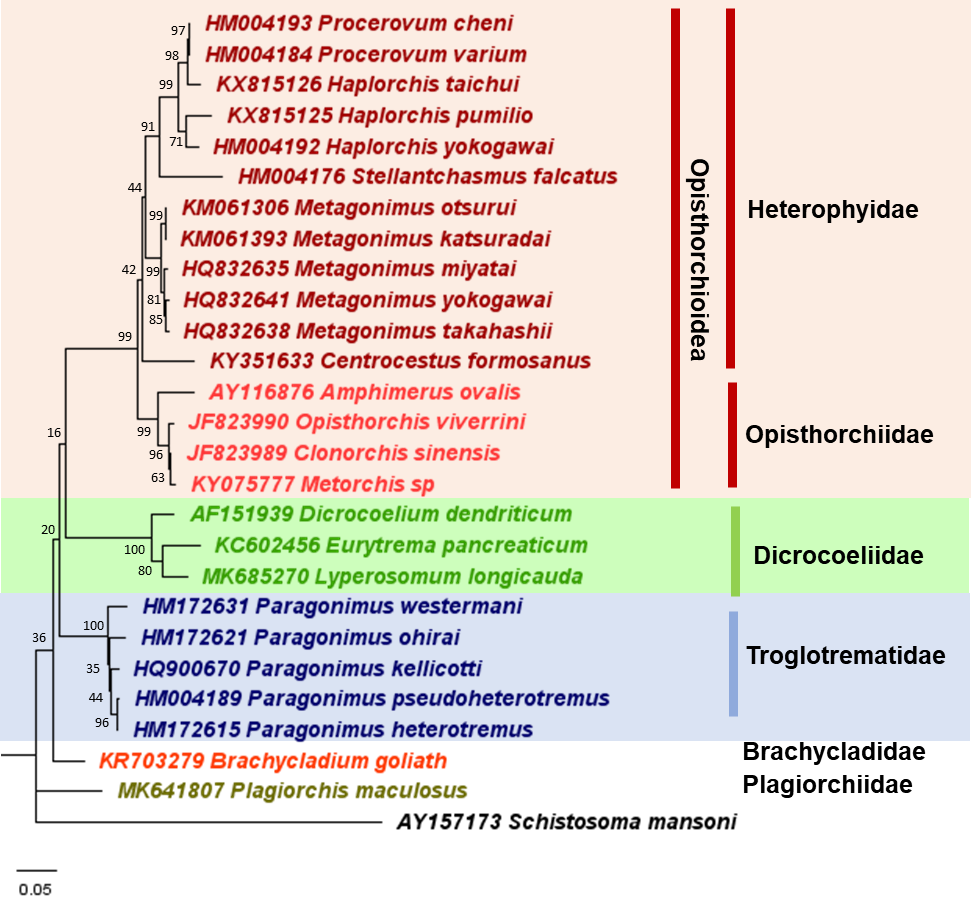
**

**Fig. S1b** Maximum likelihood phylogenetic tree (GTR+G) of the 28S rRNA gene for order Plagiorchiida

Numbers at nodes indicate bootstrap values. The superfamilies/families that were recovered as monophyletic are highlighted

**
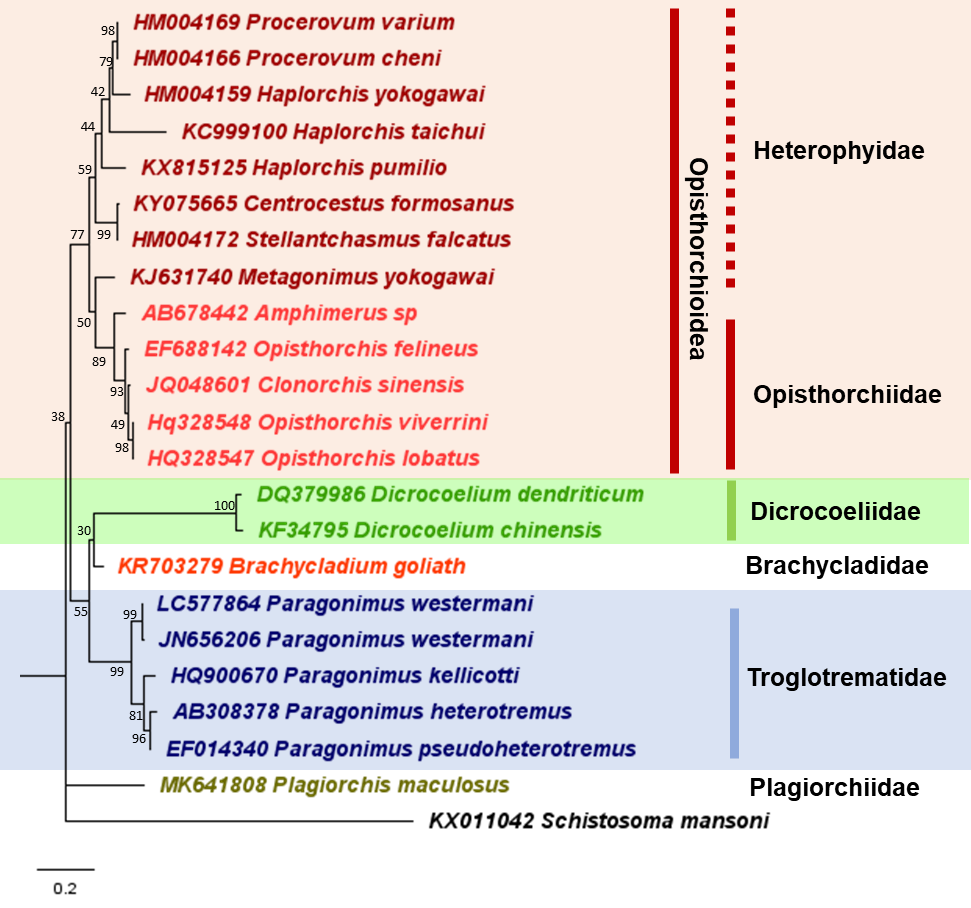
**

**Fig. S1c** Maximum likelihood phylogenetic tree (K2P+G) of the ITS2 region for order Plagiorchiida

Numbers at nodes indicate bootstrap values. The superfamilies/families that were recovered as monophyletic are highlighted

**
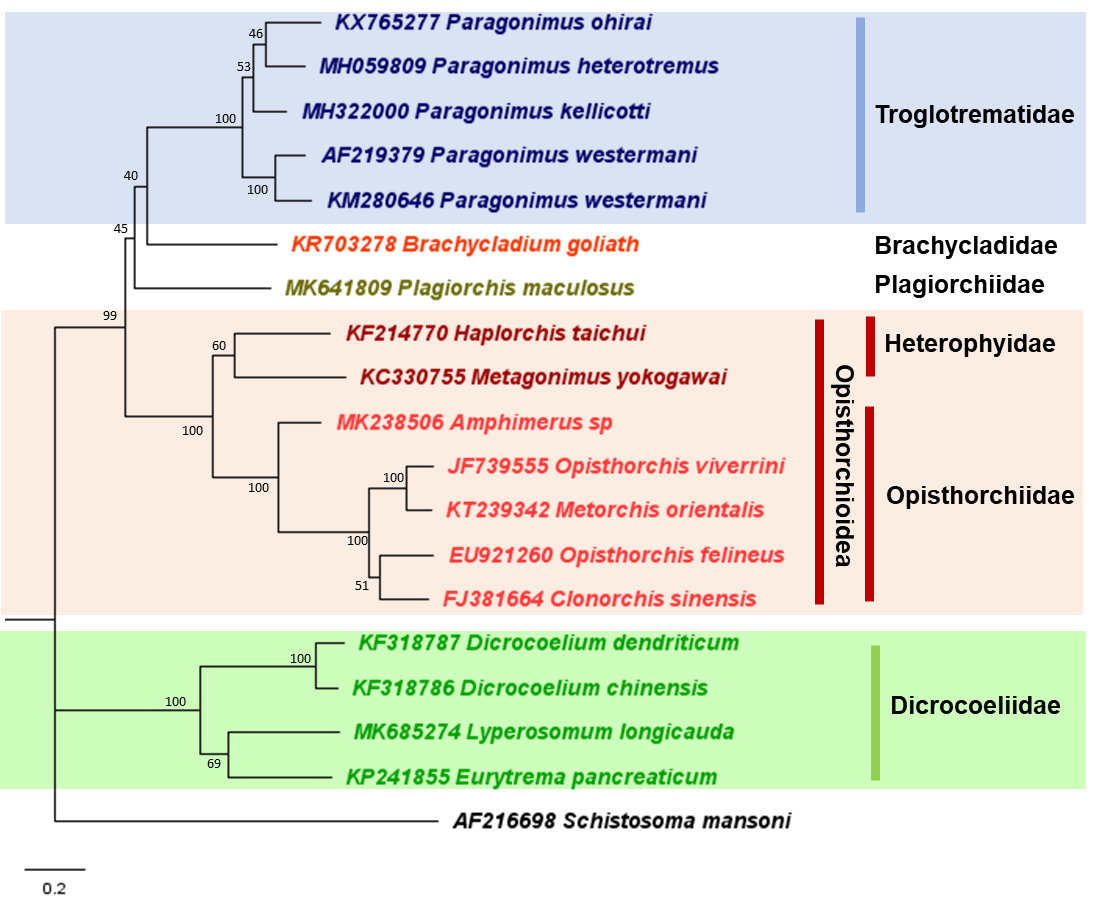
**

**Fig. S1d** Maximum likelihood phylogenetic tree (GTR+G+I) of the *COI* gene for order Plagiorchiida

Numbers at nodes indicate bootstrap values. The superfamilies/families that were recovered as monophyletic are highlighted

**
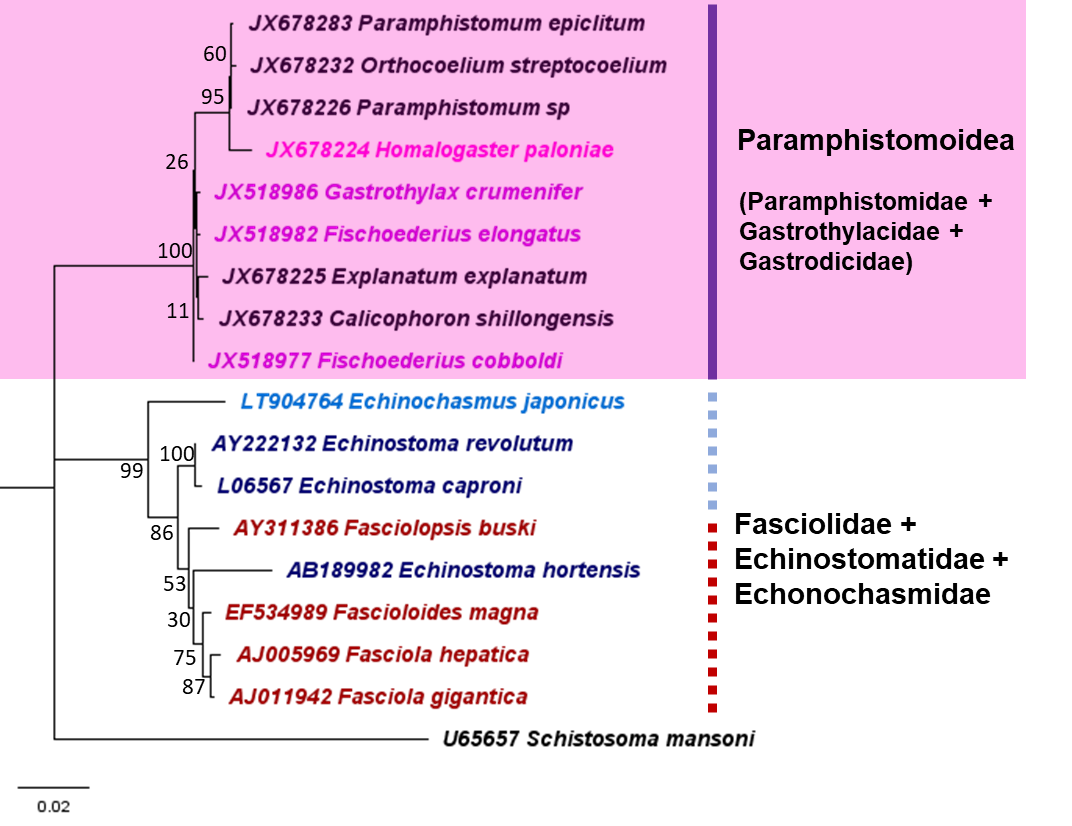
**

**Fig. S2a** Maximum likelihood phylogenetic tree (TN+G) of the 18S rRNA gene for order Echinostomida

Numbers at nodes indicate bootstrap values. The superfamilies/families that were recovered as monophyletic are highlighted

**
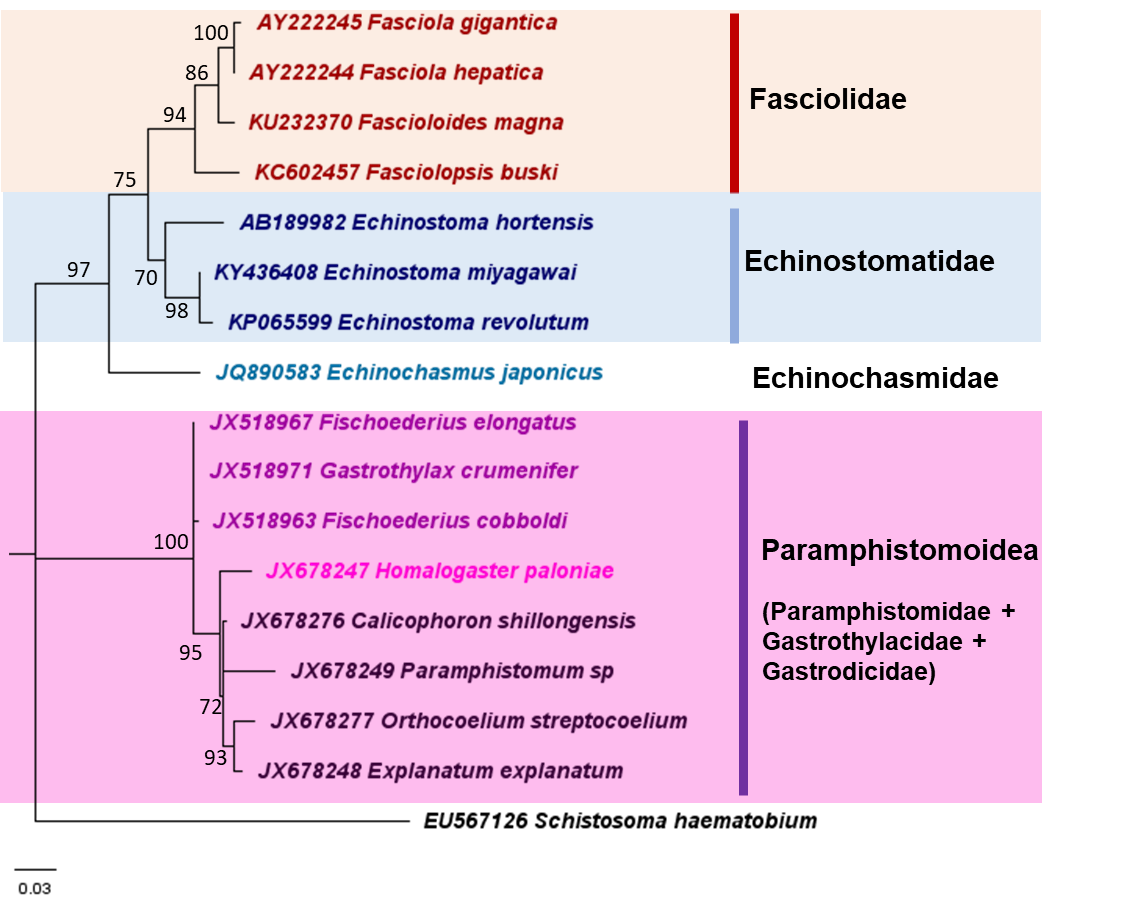
**

**Fig. S2b** Maximum likelihood phylogenetic tree (GTR+G) of the 28S rRNA gene for order Echinostomida

Numbers at nodes indicate bootstrap values. The superfamilies/families that were recovered as monophyletic are highlighted

**
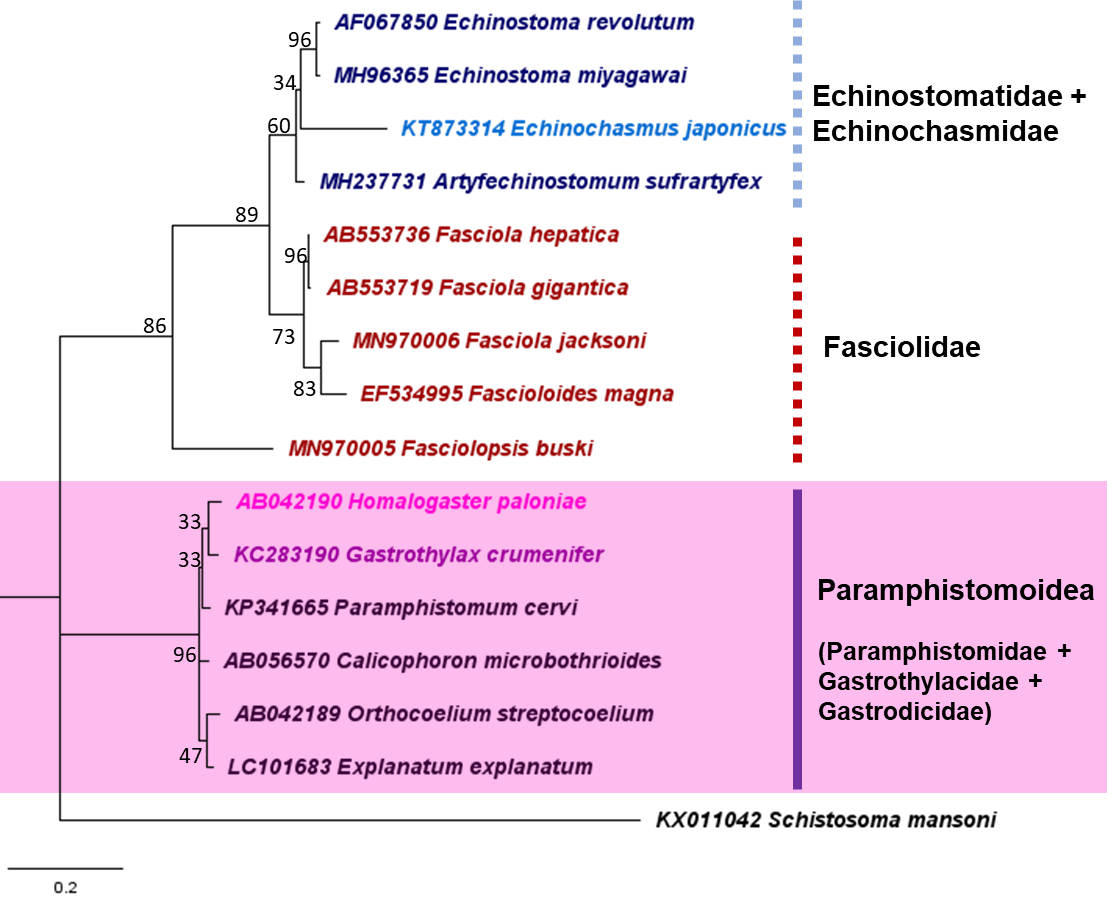
**

**Fig. S2c** Maximum likelihood phylogenetic tree (K2P+G) of the ITS2 region for order Echinostomida

Numbers at nodes indicate bootstrap values. The superfamilies/families that were recovered as monophyletic are highlighted

**
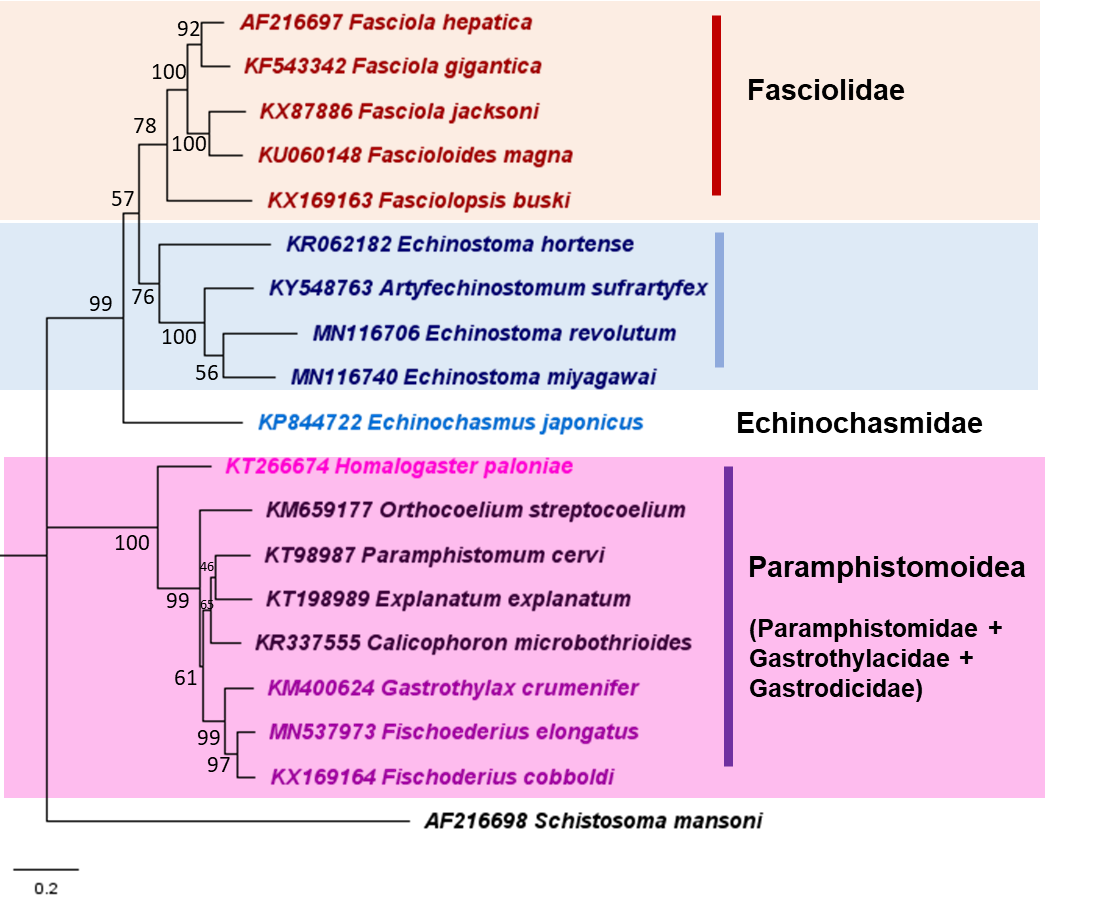
**

**Fig. S2d** Maximum likelihood phylogenetic tree (TN+G+I) of the *COI* gene for order Echinostomida

Numbers at nodes indicate bootstrap values. The superfamilies/families that were recovered as monophyletic are highlighted

**
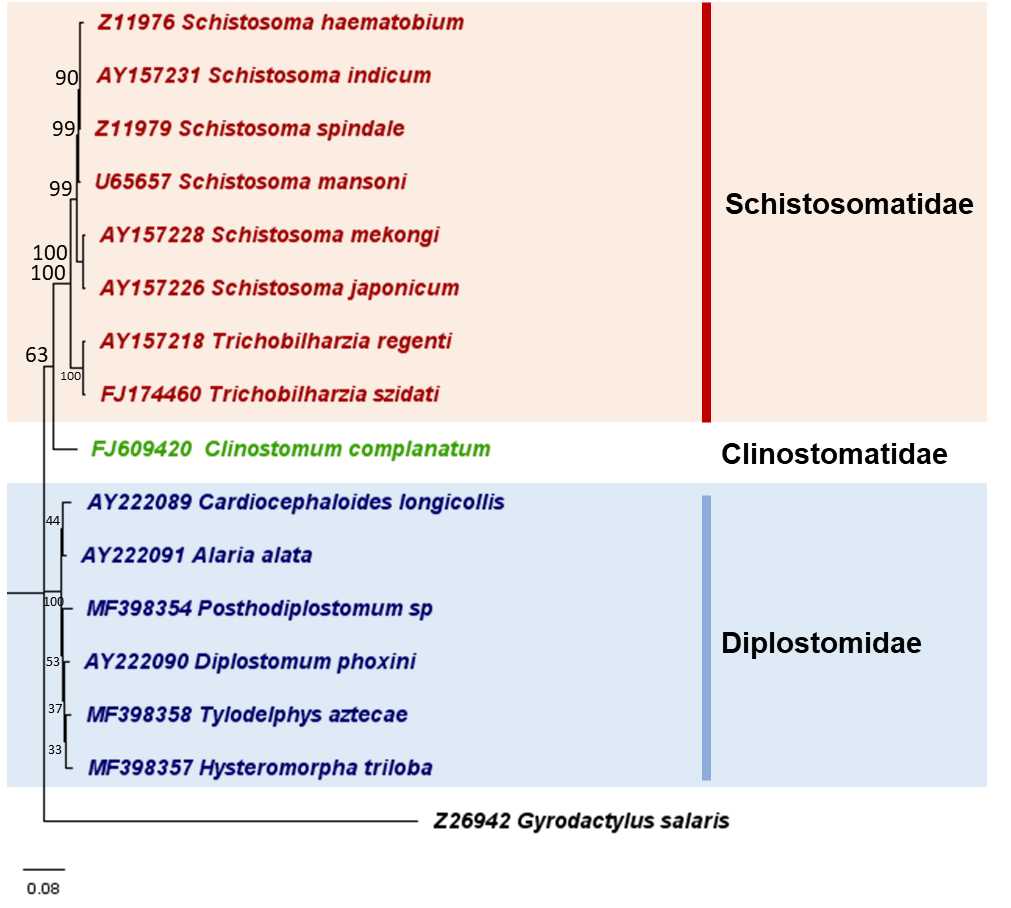
**

**Fig. S3a** Maximum likelihood phylogenetic tree (K2P+G) of the 18S rRNA gene for order Strigeida Numbers at nodes indicate bootstrap values. The families that were recovered as monophyletic are highlighted

**
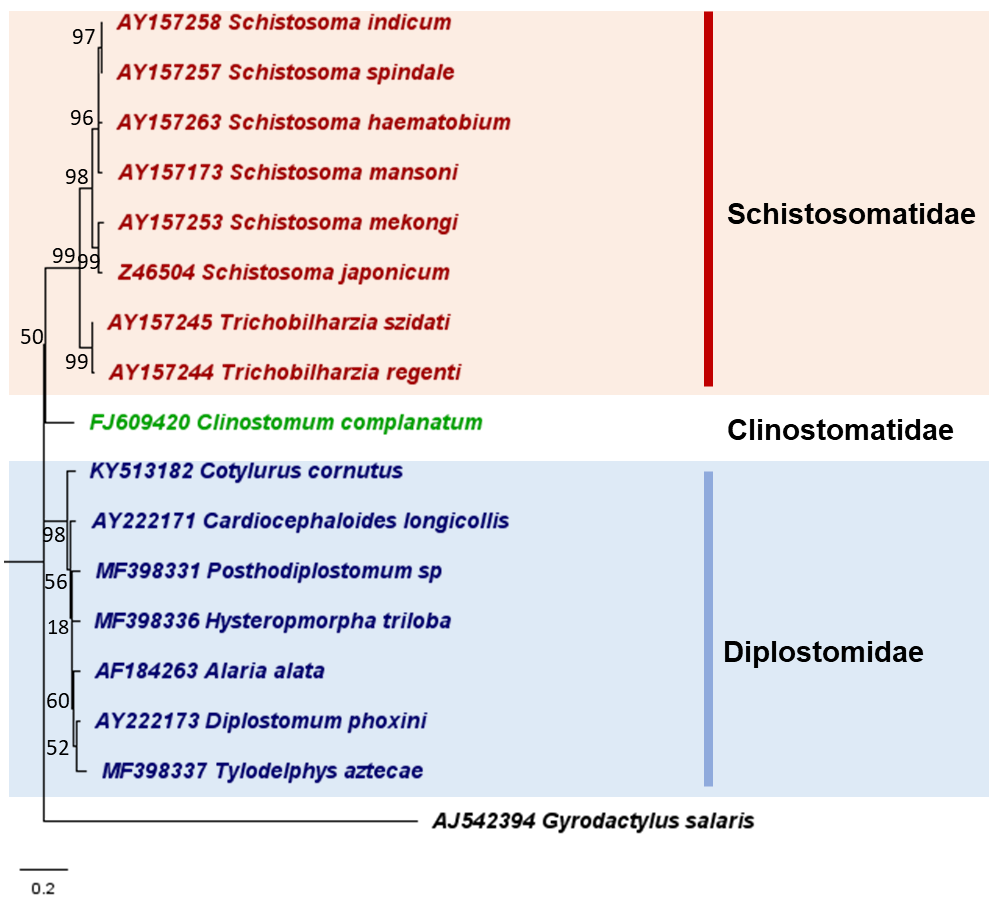
**

**Fig. S3b** Maximum likelihood phylogenetic tree (GTR+G) of the 28S rRNA gene for order Strigeida

Numbers at nodes indicate bootstrap values. The families that were recovered as monophyletic are highlighted

**
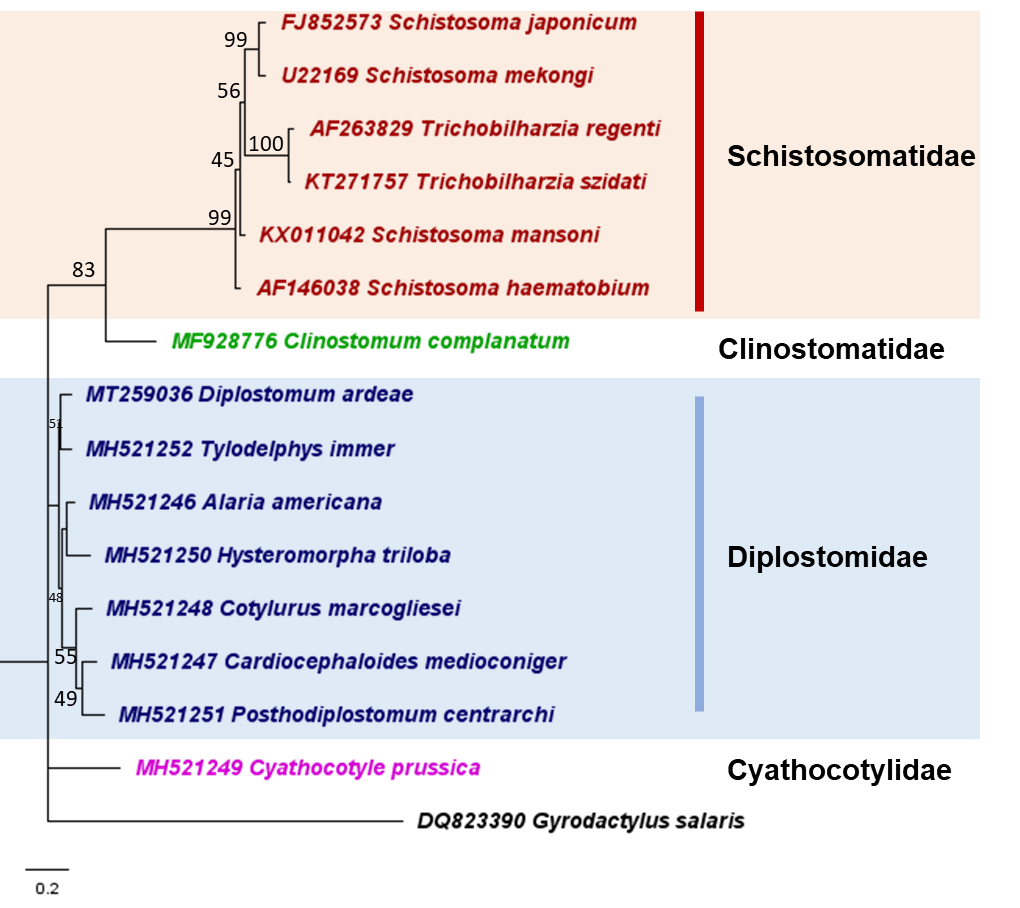
**

**Fig. S3c** Maximum likelihood phylogenetic tree (K2P+G) of the ITS2 region for order Strigeida

Numbers at nodes indicate bootstrap values. The families that were recovered as monophyletic are highlighted

**
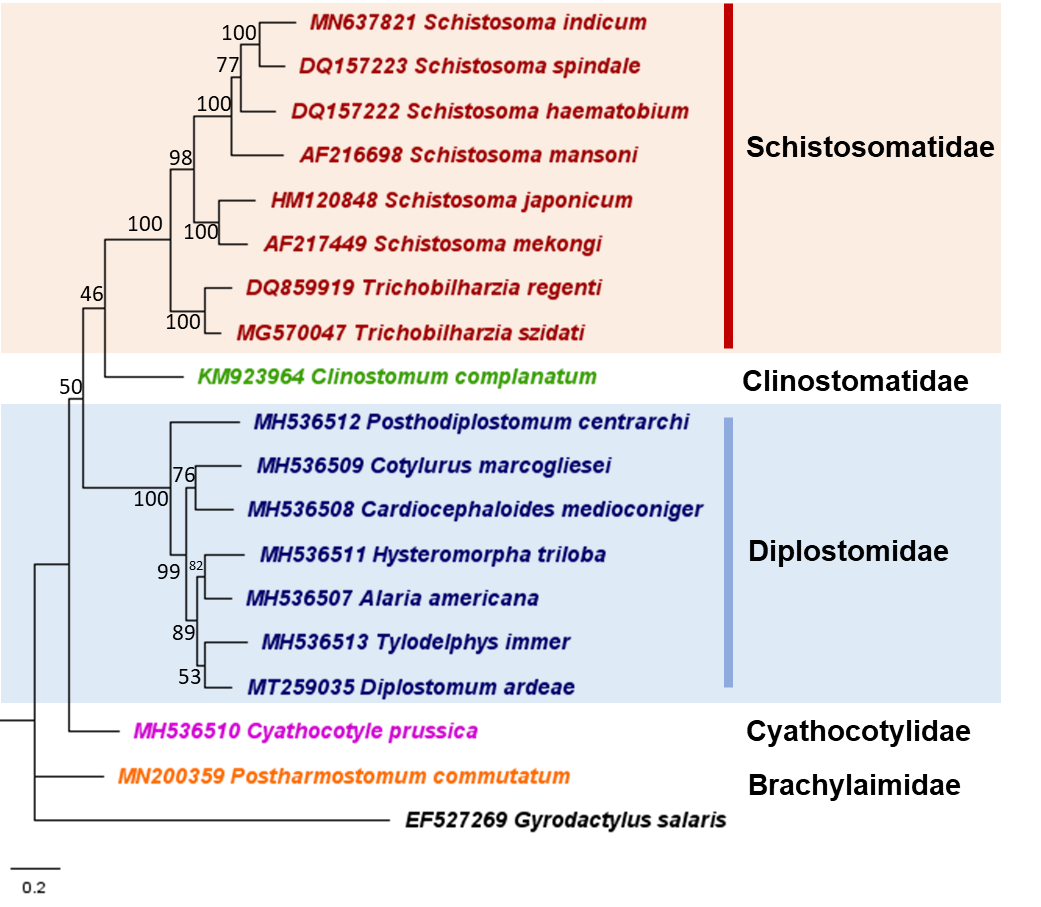
**

**Fig. S3d** Maximum likelihood phylogenetic tree (GTR+G+I) of the *COI* gene for order Strigeida

Numbers at nodes indicate bootstrap values. The families that were recovered as monophyletic are highlighted
