# Supplementary material for: The potential use of mitochondrial ribosomal genes (12S and 16S) in DNA barcoding and phylogenetic analysis of trematodes
Source: BMC Genomics. 2022 Feb 7;23:104. doi: 10.1186/s12864-022-08302-4 (PMC8822746; doi:10.1186/s12864-022-08302-4)
Supplement: Supplementary file 2 — Additional file 2: Figure S1 to S3. Maximum likelihood phylogenetic trees for the concatenated nuclear rRNA and mitochondrial rRNA genes. [file 12864_2022_8302_MOESM2_ESM.docx]

**Additional file 2:** Maximum likelihood phylogenetic trees for the concatenated nuclear rRNA and mitochondrial rRNA genes

**
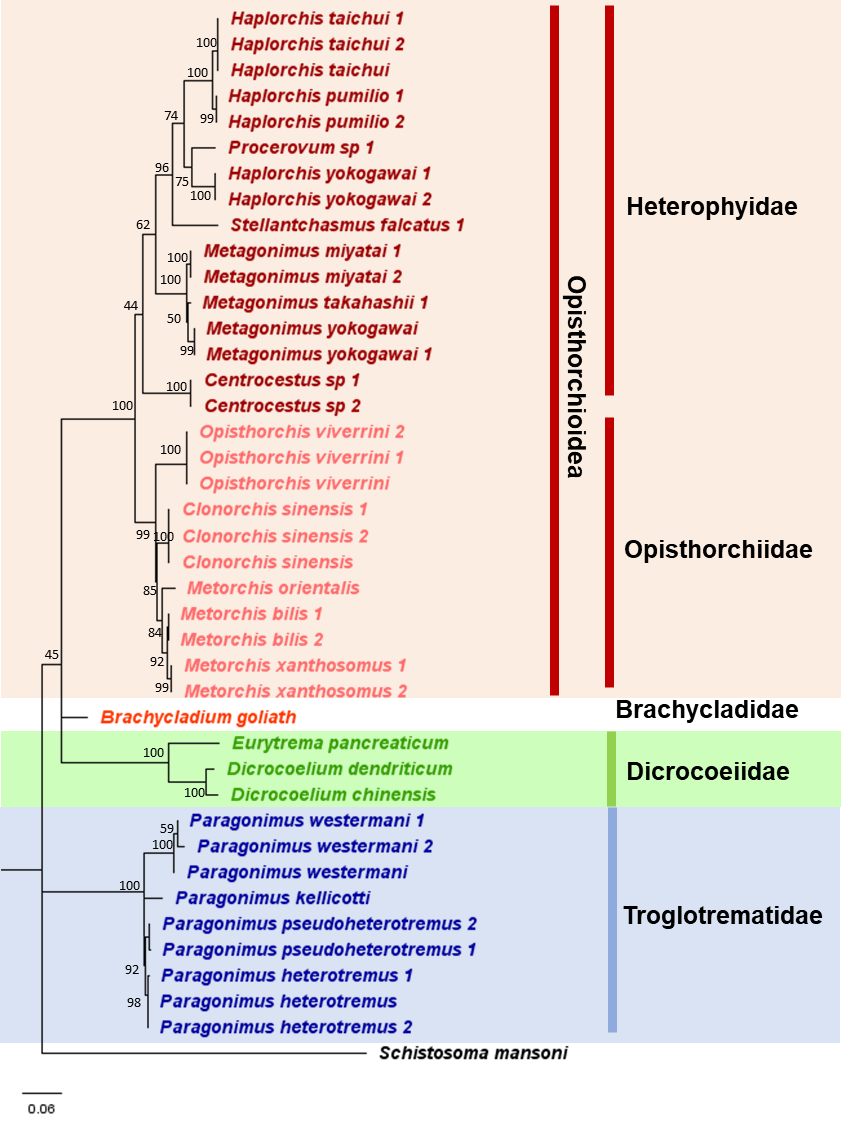
**

**Fig. S1a** Maximum likelihood phylogenetic tree (HKY+G+I) of the 18S rRNA gene with the 16S rRNA gene for order for order Plagiorchiida

Numbers at nodes indicate bootstrap values. The superfamilies/families that were recovered as monophyletic are highlighted

**
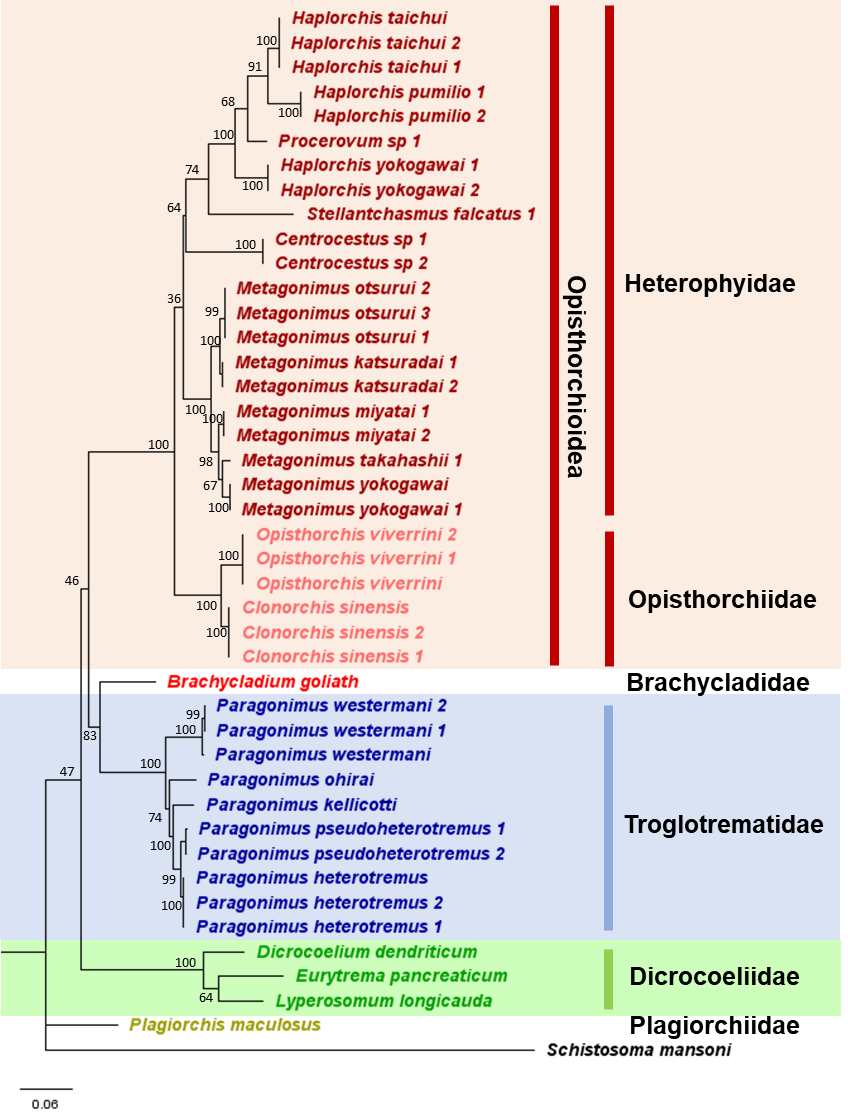
**

**Fig. S1b** Maximum likelihood phylogenetic tree (GTR+G) of the 28S rRNA gene with the 16S rRNA gene for order for order Plagiorchiida

Numbers at nodes indicate bootstrap values. The superfamilies/families that were recovered as monophyletic are highlighted

**
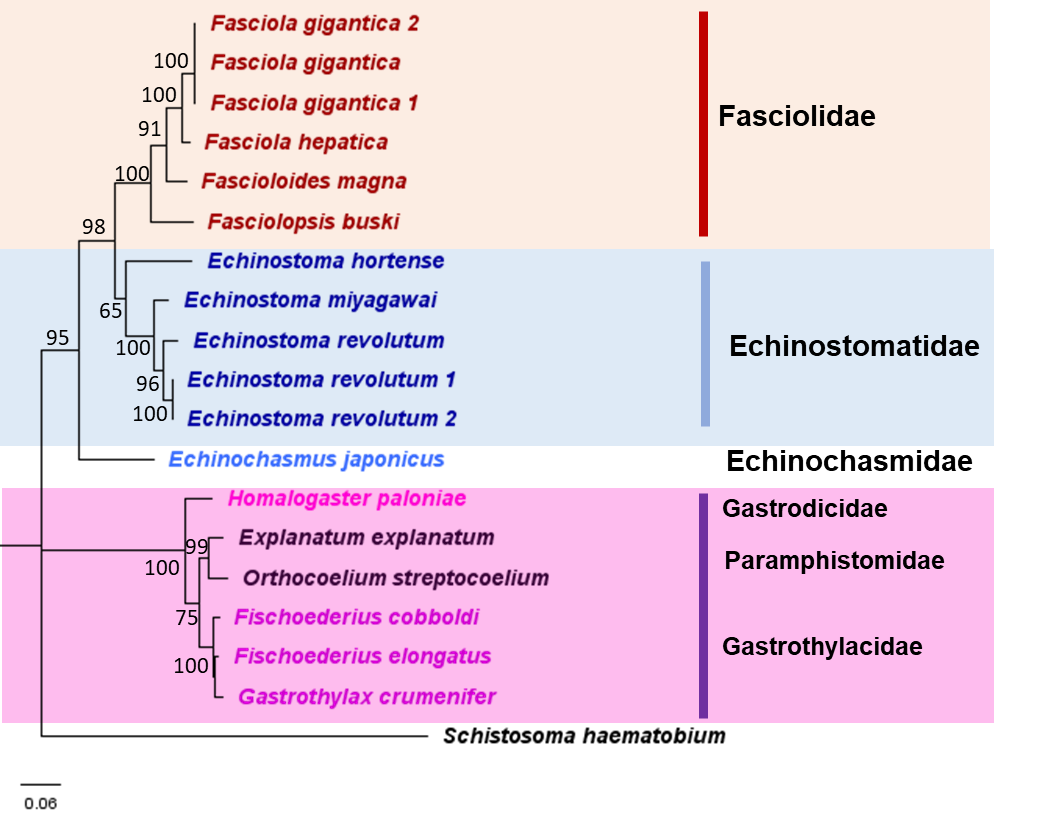
**

**Fig. S2a** Maximum likelihood phylogenetic tree (GTR+G+I) of the 28S rRNA gene with the 12S rRNA gene for order for order Echinostomida

Numbers at nodes indicate bootstrap values. The superfamilies/families that were recovered as monophyletic are highlighted

**
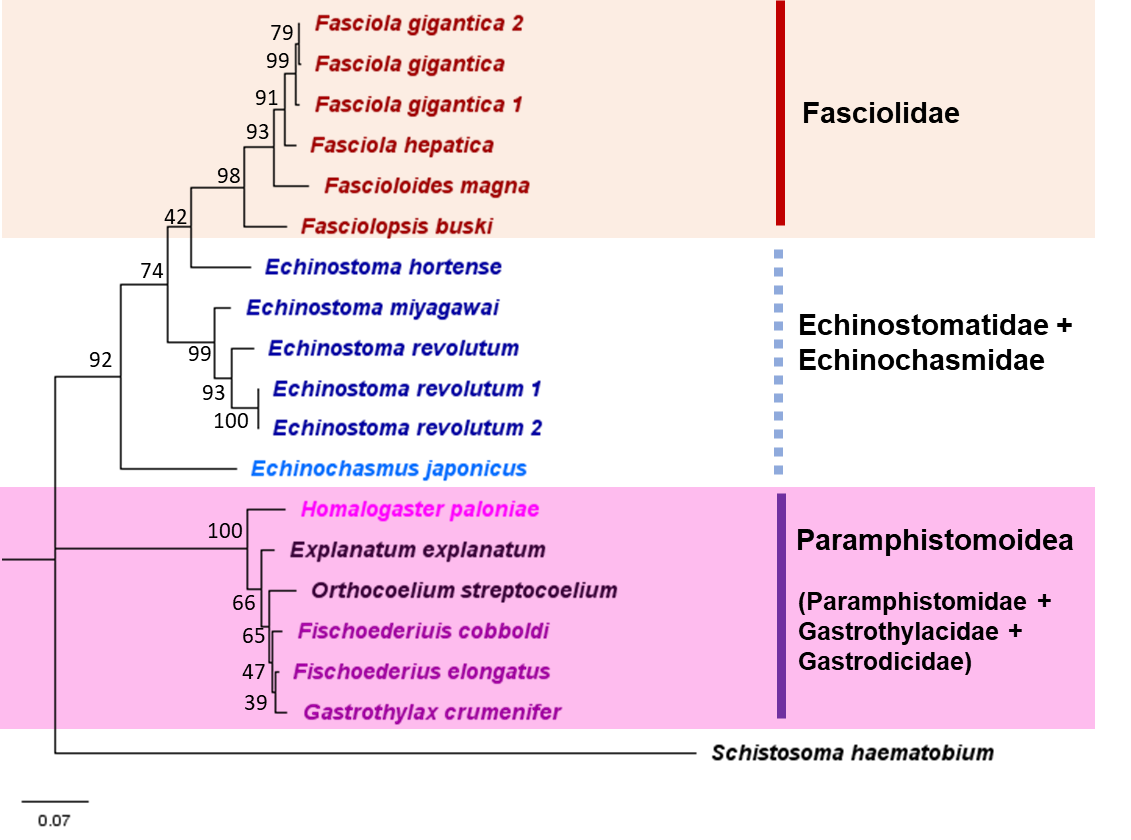
**

**Fig. S2b** Maximum likelihood phylogenetic tree (GTR+G+I) of the 28S rRNA gene with the 16S rRNA gene for order for order Echinostomida

Numbers at nodes indicate bootstrap values. The superfamilies/families that were recovered as monophyletic are highlighted

**
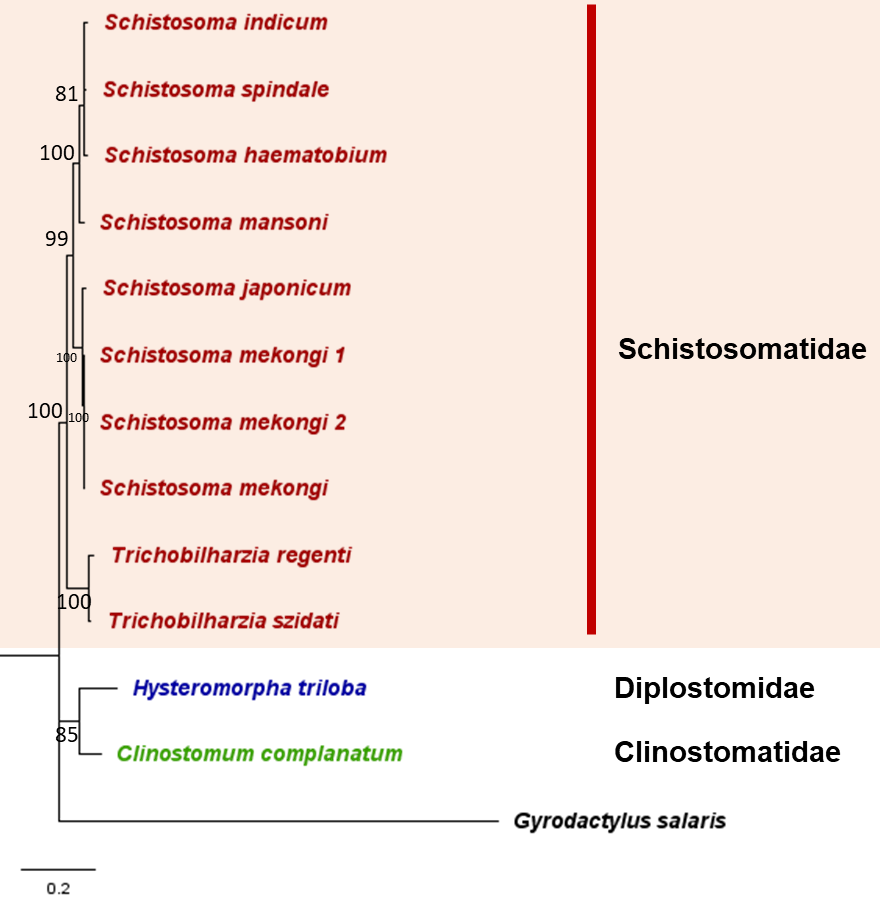
**

**Fig. S3a Maximum likelihood phylogenetic tree (GTR+G+I) of the 18S rRNA gene with the 12S rRNA gene for order Strigeida**

Numbers at nodes indicate bootstrap values. The superfamilies/families that were recovered as monophyletic are highlighted

**
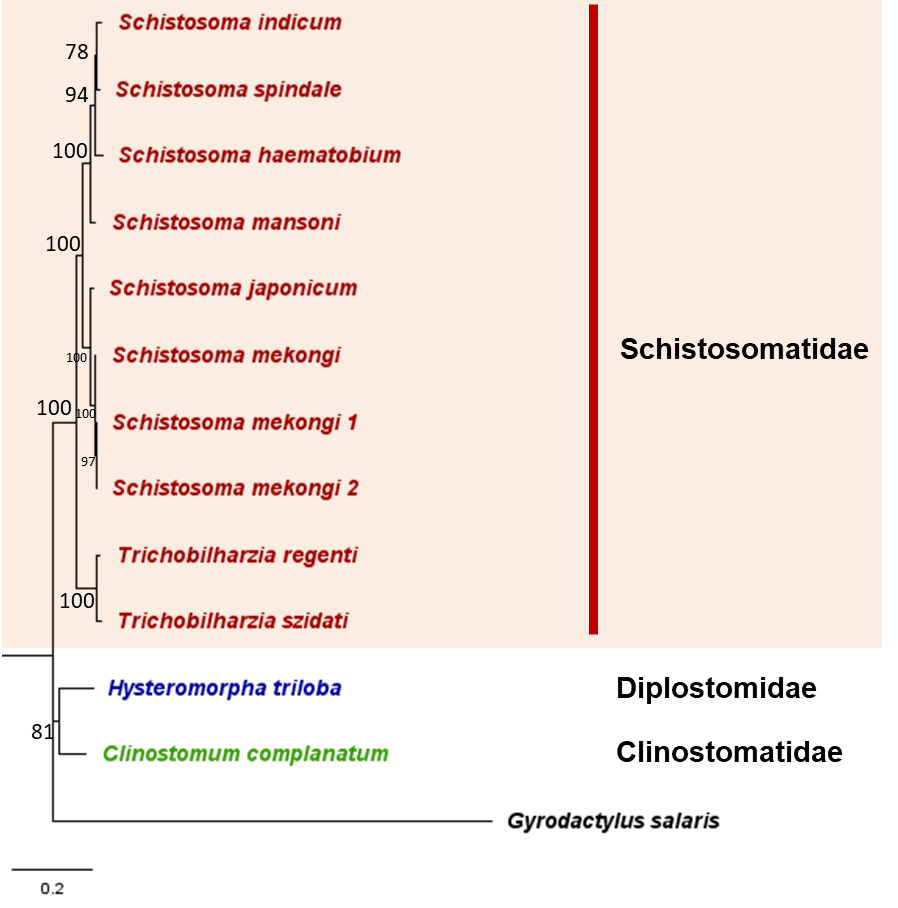
**

**Fig. S3b Maximum likelihood phylogenetic tree (GTR+G+I) of the 18S rRNA gene with the 16S rRNA gene for order Strigeida**

Numbers at nodes indicate bootstrap values. The superfamilies/families that were recovered as monophyletic are highlighted
