# Supplementary material for: The potential use of mitochondrial ribosomal genes (12S and 16S) in DNA barcoding and phylogenetic analysis of trematodes
Source: BMC Genomics. 2022 Feb 7;23:104. doi: 10.1186/s12864-022-08302-4 (PMC8822746; doi:10.1186/s12864-022-08302-4)
Supplement: Supplementary file 3 — Additional file 3: Table S1. NCBI sequences generated in this study and trematode species used for phylogenetic analysis. [file 12864_2022_8302_MOESM3_ESM.docx]

**Additional file 3: Table S1:** NCBI sequences generated in this study and trematode species used for phylogenetic analysis

| **Genetic marker** | **Taxonomic classification levels** | | | **NCBI accession number** |
| --- | --- | --- | --- | --- |
|  | **Order** | **Family** | **Species** |  |
| 12S, 16S rRNA, *COI* | Plagiorchiida | Opisthorchiidae | *Opisthorchis viverrini* | JF739555 |
|  |  |  | ***Opisthorchis viverrini*** | **MZ331639/ MZ331603** |
|  |  |  | ***Opisthorchis viverrini*** | **MZ331640/ MZ331604** |
|  |  |  | *Opisthorchis felineus* | EU921260 |
|  |  |  | ***Opisthorchis lobatus*** | **MZ331637/ MZ331601** |
|  |  |  | ***Opisthorchis lobatus*** | **MZ331638/ MZ331602** |
|  |  |  | *Clonorchis sinensis* | FJ381664 |
|  |  |  | ***Clonorchis sinensis*** | **MZ331635/ MZ331595** |
|  |  |  | ***Clonorchis sinensis*** | **MZ331636/ MZ331596** |
|  |  |  | *Metorchis orientalis* | KT239342 |
|  |  |  | ***Metorchis bilis*** | **MZ331641/ MZ331597** |
|  |  |  | ***Metorchis bilis*** | **MZ331642/ MZ331598** |
|  |  |  | ***Metorchis xantosomus*** | **MZ331643/ MZ331659** |
|  |  |  | ***Metorchis xantosomus*** | **MZ331660** |
|  |  |  | *Amphimerus* sp. | MK238506 |
|  |  | Heterophyidae | *Haplorchis taichui* | KF214770 |
|  |  |  | ***Haplorchis taichui*** | **MZ331646/ MZ331605** |
|  |  |  | ***Haplorchis taichui*** | **MZ331606** |
|  |  |  | ***Haplorchis pumilio*** | **MZ331644/ MZ331607** |
|  |  |  | ***Haplorchis pumilio*** | **MZ331645/ MZ331608** |
|  |  |  | ***Haplorchis yokogawai*** | **MZ331647/ MZ331609** |
|  |  |  | ***Haplorchis yokogawai*** | **MZ331610** |
|  |  |  | ***Procerovum* sp.** | **MZ331648/ MZ331611** |
|  |  |  | ***Stellantchasmus falcatus*** | **MZ331649/ MZ331612** |
|  |  |  | ***Stellantchasmus falcatus*** | **MZ331650** |
|  |  |  | ***Centrocestus* sp.** | **MZ331651/ MZ331613** |
|  |  |  | ***Centrocestus* sp.** | **MZ331652/ MZ331614** |
|  |  |  | *Metagonimus yokogawai* | KC330755 |
|  |  |  | ***Metagonimus yokogawai*** | **MZ331661/ MZ331621** |
|  |  |  | ***Metagonimus yokogawai*** | **MZ331662** |
|  |  |  | ***Metagonimus takahashii*** | **MZ331657/ MZ331620** |
|  |  |  | ***Metagonimus miyatai*** | **MZ331655/ MZ331618** |
|  |  |  | ***Metagonimus miyatai*** | **MZ331656/ MZ331619** |
|  |  |  | ***Metagonimus katsuradai*** | **MZ331653/ MZ331616** |
|  |  |  | ***Metagonimus katsuradai*** | **MZ331654/ MZ331617** |
|  |  |  | ***Metagonimus otsuri*** | **MZ331658/ MZ331622** |
|  |  |  | ***Metagonimus otsuri*** | **MZ331659/ MZ331623** |
|  |  |  | ***Metagonimus otsuri*** | **MZ331660/ MZ331624** |
|  |  | Cryptogonimidae | ***Exorchis oviformis*** | **MZ331663/ MZ331615** |
|  |  |  | ***Exorchis oviformis*** | **MZ331664** |
|  |  | Troglotrematidae | *Paragonimus westermani* | AF219379 |
|  |  |  | *Paragonimus westermani* | KM280646 |
|  |  |  | ***Paragonimus westermani*** | **MZ331667/ MZ345702** |
|  |  |  | ***Paragonimus westermani*** | **MZ331668/ MZ345703** |
|  |  |  | *Paragonimus heterotremus* | MH059809 |
|  |  |  | ***Paragonimus heterotremus*** | **MZ331665/ MZ345698** |
|  |  |  | ***Paragonimus heterotremus*** | **MZ331666/ MZ345699** |
|  |  |  | ***Paragonimus pseudoheterotremus*** | **MZ331669/ MZ345700** |
|  |  |  | ***Paragonimus pseudoheterotremus*** | **MZ331670/ MZ345701** |
|  |  |  | *Paragonimus kellicotti* | MH322000 |
|  |  |  | *Paragonimus ohirai* | KX765277 |
|  |  | Brachycladidae | *Brachycladium goliath* | KR703278 |
|  |  | Plagiorchiidae | *Plagiorchis maculosus* | MK641809 |
|  |  | Dicrocoeliidae | *Dicrocoelium dendriticum* | KF318787 |
|  |  |  | *Dicrocoelium chinensis* | KF318786 |
|  |  |  | *Lyperosomum longicauda* | MK685274 |
|  |  |  | *Eurytrema pancreaticum* | KP241855 |
|  |  |  | ***Eurytrema* sp.** | **MZ331671/ MZ345704** |
|  |  |  | ***Eurytrema* sp.** | **MZ331672/ MZ435705** |
|  |  |  | ***Eurytrema* sp.** | **MZ331673** |
|  | Echinostomida | Echinostomatidae | *Echinostoma revolutum* | MN116706 |
|  |  |  | ***Echinostoma revolutum*** | **MZ331674/ MZ331625** |
|  |  |  | ***Echinostoma revolutum*** | **MZ331675/ MZ331626** |
|  |  |  | *Echinostoma miyagawai* | MN116740 |
|  |  |  | *Echinostoma caproni* | AP017706 |
|  |  |  | *Echinostoma hortense* | KR062182 |
|  |  |  | *Artyfechionostomum sufrartyfex* | KY548763 |
|  |  | Echinochasmidae | *Echinochamus japonicus* | KP844722 |
|  |  | Fasciolidae | *Fasciola hepatica* | AF216697 |
|  |  |  | *Fasciola gigantica* | KF543342 |
|  |  |  | ***Fasciola gigantica*** | **MZ331676/ MZ331627** |
|  |  |  | ***Fasciola gigantica*** | **MZ331677/ MZ331628** |
|  |  |  | *Fasciola jacksoni* | KX787886 |
|  |  |  | *Fasciolopsis buski* | KX169163 |
|  |  |  | *Fascioloides magna* | KU060148 |
|  |  | Gastrodiscidae | *Homalogaster paloniae* | KT266674 |
|  |  | Paramphistomidae | *Explanatum explanatum* | KT198989 |
|  |  |  | *Paramphistomum cervi* | KT198987 |
|  |  |  | *Calicophoron microbothrioides* | KR337555 |
|  |  |  | *Orthocoelium streptocoelium* | KM659177 |
|  |  | Gastrothylacidae | *Gastrothylax crumenifer* | KM400624 |
|  |  |  | ***Gastrothylax* sp.** | **MZ331678/ MZ331629** |
|  |  |  | ***Gastrothylax* sp.** | **MZ331679/ MZ331630** |
|  |  |  | *Fischoederrius elongatus* | MN537973 |
|  |  |  | *Fischoederius cobboldi* | KX169164 |
|  | Strigeida | Diplostomidae | *Hysteromorpha triloba* | MH536511 |
|  |  |  | *Diplostomum ardeae* | MT259035 |
|  |  |  | *Tylodelphys immer* | MH536513 |
|  |  |  | *Posthodiplostomum centrarchi* | MH536512 |
|  |  |  | *Cotylurus marcogliesei* | MH536509 |
|  |  |  | *Cardiocephaloides medioconiger* | MH536508 |
|  |  |  | *Alaria americana* | MH536507 |
|  |  |  | *Postharmostomum commutatus* | MN200359 |
|  |  | Cyanthocotylidae | *Cyathocotyle prussica* | MH536510 |
|  |  | Schistosomatidae | *Schistosoma haematobium* | DQ157222 |
|  |  |  | *Schistosoma indicum* | MN637821 |
|  |  |  | *Schistosoma japonicum* | HM120848 |
|  |  |  | *Schistosoma mekongi* | AF217449 |
|  |  |  | ***Schistosoma mekongi*** | **MZ331682/ MZ331633** |
|  |  |  | ***Schistosoma mekongi*** | **MZ331683/ MZ331634** |
|  |  |  | *Schistosoma spindale* | DQ157223 |
|  |  |  | *Schistosoma mansoni* | AF216698 |
|  |  |  | *Trichobilharzia regent* | DQ859919 |
|  |  |  | *Trichobilharzia szidati* | MG570047 |
|  |  | Clinostomidae | *Clinostomum complanatum* | KM923964 |
|  |  |  | ***Clinostomum* sp.** | **MZ331680/ MZ331631** |
|  |  |  | ***Clinostomum* sp.** | **MZ331681/ MZ331632** |
|  | Outgroup |  | *Gyrodactylus salaris* | EF527269 |
| **18S rRNA gene** | Plagiorchiida | Opisthorchiidae | *Opisthorchis viverrini* | JF823987 |
|  |  |  | *Clonorchis sinensis* | JF314770 |
|  |  |  | *Metorchis orientalis* | JF314771 |
|  |  |  | *Metorchis bilis* | KT740964 |
|  |  |  | *Metorchis xanthosomus* | KT740962 |
|  |  |  | *Amphimerus ovalis* | AY222121 |
|  |  | Heterophyidae | *Haplorchis taichui* | HM004213 |
|  |  |  | *Haplorchis pumilio* | HM004196 |
|  |  |  | *Haplorchis yokogawai* | HM004208 |
|  |  |  | *Procerovum cheni* | HM004212 |
|  |  |  | *Procerovum varium* | HM004205 |
|  |  |  | *Stellantchasmus falcatus* | HM004209 |
|  |  |  | *Centrocestus formosanus* | HQ874608 |
|  |  |  | *Metagonimus yokogawai* | HQ832632 |
|  |  |  | *Metagonimus takahashii* | HQ832629 |
|  |  |  | *Metagonimus miyatai* | HQ832626 |
|  |  | Troglotrematidae | *Paragonimus westermani* | AJ287556 |
|  |  |  | *Paragonimus heterotremus* | LT855188 |
|  |  |  | *Paragonimus pseudoheterotremus* | HM004210 |
|  |  |  | *Paragonimus kellicotti* | HQ900670 |
|  |  | Brachycladidae | *Brachycladium goliath* | KR703279 |
|  |  | Dicrocoeliidae | *Dicrocoelium dendriticum* | Y11236 |
|  |  |  | *Dicrocoelium orientalis* | EF547131 |
|  |  |  | *Eurytrema pancreaticum* | DQ401034 |
|  | Echinostomida | Echinostomatidae | *Echinostoma revolutum* | AY222132 |
|  |  |  | *Echinostoma caproni* | L06567 |
|  |  |  | *Echinostoma hortensis* | AB189982 |
|  |  | Echinochasmidae | *Echinochasmus japonicus* | LT904764 |
|  |  | Fasciolidae | *Fasciola hepatica* | AJ004969 |
|  |  |  | *Fasciola gigantica* | AJ011942 |
|  |  |  | *Fasciolopsis buski* | AY311386 |
|  |  |  | *Fascioloides magna* | EF534989 |
|  |  | Gastrodiscidae | *Homalogaster paloniae* | JX678224 |
|  |  | Paramphistomidae | *Explanatum explanatum* | JX678225 |
|  |  |  | *Paramphistomum sp.* | JX678226 |
|  |  |  | *Paramphistomum epiclitum* | JX678283 |
|  |  |  | *Calicophoron shillongensis* | JX678233 |
|  |  |  | *Orthocoelium streptocoelium* | JX678232 |
|  |  | Gastrothylacidae | *Gastrothylax crumenifer* | JX518986 |
|  |  |  | *Fischoederius elongatus* | JX518982 |
|  |  |  | *Fischoederius cobboldi* | JX518977 |
|  | Strigeida | Diplostomidae | *Hysteromorpha triloba* | MF398357 |
|  |  |  | *Diplostomum phoxini* | AY222090 |
|  |  |  | *Tylodelphys aztecae* | MF398358 |
|  |  |  | *Posthodiplostomum sp.* | MF398354 |
|  |  |  | *Cardiocephaloides longicollis* | AY222089 |
|  |  |  | *Alaria alata* | AY222091 |
|  |  | Schistosomatidae | *Schistosoma haematobium* | Z11976 |
|  |  |  | *Schistosoma indicum* | AY157231 |
|  |  |  | *Schistosoma mekongi* | AY157228 |
|  |  |  | *Schistosoma japonicum* | AY157226 |
|  |  |  | *Schistosoma mansoni* | *U65657* |
|  |  |  | *Schistosoma spindale* | Z11979 |
|  |  |  | *Trichobilharzia regenti* | AY157218 |
|  |  |  | *Trichobilharzia szidati* | FJ174460 |
|  |  | Clinostomidae | *Clinostomum complanatum* | FJ609420 |
|  | Outgroup |  | *Gyrodactylus salaris* | Z26942 |
| **28S rRNA gene** | Plagiorchiida | Opisthorchiidae | *Opisthorchis viverrini* | JF823990 |
|  |  |  | *Clonorchis sinensis* | JF823989 |
|  |  |  | *Metorchis sp.* | KY075777 |
|  |  |  | *Amphimerus ovalis* | AY116876 |
|  |  | Heterophyidae | *Haplorchis taichui* | KX815126 |
|  |  |  | *Haplorchis pumilio* | KX815125 |
|  |  |  | *Haplorchis yokogawai* | HM004192 |
|  |  |  | *Procerovum cheni* | HM004193 |
|  |  |  | *Procerovum varium* | HM004184 |
|  |  |  | *Stellantchasmus falcatus* | HM004176 |
|  |  |  | *Centrocestus formosanus* | KY351633 |
|  |  |  | *Metagonimus yokogawai* | HQ832641 |
|  |  |  | *Metagonimus takahashii* | HQ832638 |
|  |  |  | *Metagonimus miyatai* | HQ832635 |
|  |  |  | *Metagonimus otsurui* | KM061396 |
|  |  |  | *Metagonimus katsuradai* | KM061393 |
|  |  | Troglotrematidae | *Paragonimus westermani* | HM172631 |
|  |  |  | *Paragonimus heterotremus* | HM172615 |
|  |  |  | *Paragonimus pseudoheterotremus* | HM004189 |
|  |  |  | *Paragonimus ohirai* | HM172621 |
|  |  |  | *Paragonimus kellicotti* | HQ900670 |
|  |  | Brachycladidae | *Brachycladium goliath* | KR703279 |
|  |  | Plagiorchiidae | *Plagiorchis maculosus* | MK641807 |
|  |  | Dicrocoeliidae | *Dicrocoelium dendriticum* | AF151939 |
|  |  |  | *Eurytrema pancreaticum* | KC602456 |
|  |  |  | *Lyperosomum longicauda* | MK685270 |
|  | Echinostomida | Echinostomatidae | *Echinostoma revolutum* | KP065599 |
|  |  |  | *Echinostoma miyagawai* | KY436408 |
|  |  |  | *Echinostoma hortensis* | AB189982 |
|  |  | Echinochasmidae | *Echinochasmus japonicus* | JQ890583 |
|  |  | Fasciolidae | *Fasciola hepatica* | AY222244 |
|  |  |  | *Fasciola gigantica* | AY222245 |
|  |  |  | *Fascioloides magna* | KU232370 |
|  |  |  | *Fasciolopsis buski* | KC602457 |
|  |  | Gastrodiscidae | *Homalogaster paloniae* | JX678247 |
|  |  | Paramphistomidae | *Orthocoelium streptocoelium* | JX678277 |
|  |  |  | *Explanatum explanatum* | JX678248 |
|  |  |  | *Calicophoron shillongensis* | JX678276 |
|  |  |  | *Paramphistomum sp.* | JX678249 |
|  |  | Gastrothylacidae | *Gastrothylax crumenifer* | JX518971 |
|  |  |  | *Fischoederius elongatus* | JX518967 |
|  |  |  | *Fischoederius cobboldi* | JX518963 |
|  | Strigeida | Diplostomidae | *Hysteromorpha triloba* | MF398336 |
|  |  |  | *Diplostomum phoxini* | AY222173 |
|  |  |  | *Tylodelphys aztecae* | MF398337 |
|  |  |  | *Posthodiplostomum sp.* | MF398331 |
|  |  |  | *Cotylurus cornutus* | KY513182 |
|  |  |  | *Cardiocephaloides longicollis* | AY222171 |
|  |  |  | *Alaria alata* | AF184263 |
|  |  | Schistosomatidae | *Schistosoma indicum* | AY157258 |
|  |  |  | *Schistosoma spindale* | AY157257 |
|  |  |  | *Schistosoma haematobium* | AY157263 |
|  |  |  | *Schistosoma mansoni* | AY157173 |
|  |  |  | *Schistosoma mekongi* | AY157253 |
|  |  |  | *Schistosoma japonicum* | Z46504 |
|  |  |  | *Trichobilharzia szidati* | AY157245 |
|  |  |  | *Trichobilharzia regenti* | AY157244 |
|  |  | Clinostomidae | *Clinostomum complanatum* | FJ609420 |
|  | Outgroup |  | *Gyrodactylus salaris* | AJ542394 |
| **ITS2** | Plagiorchiida | Opisthorchiidae | *Opisthorchis viverrini* | HQ328548 |
|  |  |  | *Opisthorchis lobatus* | HQ328547 |
|  |  |  | *Opisthorchis felineus* | EF688142 |
|  |  |  | *Clonorchis sinensis* | JQ048601 |
|  |  |  | *Amphimerus sp.* | AB678442 |
|  |  | Heterophyidae | *Haplorchis taichui* | KC999100 |
|  |  |  | *Haplorchis pumilio* | KX815125 |
|  |  |  | *Haplorchis yokogawai* | HM004159 |
|  |  |  | *Procerovum varium* | HM004169 |
|  |  |  | *Procerovum cheni* | HM004166 |
|  |  |  | *Stellantchasmus falcaatus* | HM004172 |
|  |  |  | *Centrocestus formosanus* | KY075665 |
|  |  |  | *Metagonimus yokogawai* | KJ631740 |
|  |  | Troglotrematidae | *Paragonimus westermani* | LC577864 |
|  |  |  | *Paragonimus westermani* | JN656206 |
|  |  |  | *Paragonimus heterotremus* | AB308378 |
|  |  |  | *Paragonimus pseudoheterotremus* | EF014340 |
|  |  |  | *Paragonimus kellicotti* | HQ900670 |
|  |  | Brachycladidae | *Brachycladium goliath* | KR703279 |
|  |  | Plagiorchiidae | *Plagiorchis maculosus* | MK641808 |
|  |  | Dicrocoeliidae | *Dicrocoelium dendriticum* | DQ379986 |
|  |  |  | *Dicrocoelium chinensis* | KF734795 |
|  | Echinostomida | Echinostomatidae | *Echinostoma revolutum* | AF067850 |
|  |  |  | *Echinostoma miyagawai* | MH796365 |
|  |  |  | *Artyfechinostomum sufrartyfex* | MH237731 |
|  |  | Echinochasmidae | *Echinochasmus japonicus* | KT873314 |
|  |  | Fasciolidae | *Fasciola hepatica* | AB553736 |
|  |  |  | *Fasciola gigantica* | AB553719 |
|  |  |  | *Fasciola jacksoni* | MN970006 |
|  |  |  | *Fascioloides magna* | EF534995 |
|  |  |  | *Fasciolopsis buski* | MN970005 |
|  |  | Gastrodiscidae | *Homalogaster paloniae* | AB042190 |
|  |  | Paramphistomidae | *Orthocoelium streptocoelium* | AB042189 |
|  |  |  | *Calicophoron microbothrioides* | AB056570 |
|  |  |  | *Paramphistomum cervi* | KP341665 |
|  |  |  | *Explanatum explanatum* | LC101683 |
|  |  | Gastrothylacidae | *Gasstrothylax crumenifer* | KC283190 |
|  | Strigeida | Diplostomidae | *Hysteromorpha triloba* | MH521250 |
|  |  |  | *Diplostomum ardeae* | MT259036 |
|  |  |  | *Tylodelphys immer* | MH521252 |
|  |  |  | *Posthodiplostomum centrarchi* | MH521251 |
|  |  |  | *Cotylurus marcogliesei* | MH521248 |
|  |  |  | *Cardiocephaloides medioconiger* | MH521247 |
|  |  |  | *Alaria americana* | MH521246 |
|  |  | Cyanthocotylidae | *Cyathocotyle prussica* | MH521249 |
|  |  | Schistosomatidae | *Schistosoma haematobium* | AF146038 |
|  |  |  | *Schistosoma japonicum* | FJ852573 |
|  |  |  | *Schistosoma mekongi* | U22169 |
|  |  |  | *Schhistosoma mansoni* | KX011042 |
|  |  |  | *Trichobilharzia regenti* | AF263829 |
|  |  |  | *Trichobilharzia szidati* | KT271757 |
|  |  | Clinostomidae | *Clinostomum complanatum* | MF928776 |
|  | Outgroup |  | *Gyrodactylus salaris* | DQ823390 |

NCBI accession numbers in **bold** are the partial 12S rRNA gene sequences generated in this study, and numbers in **bold** and underlined are the partial 16S rRNA gene sequences generated in this study.
